# Age‐Related Alterations in Hippocampal Microstructure Quantified Using High‐Gradient Diffusion MRI (dMRI) in an Unfolded Hippocampal Space

**DOI:** 10.1111/acel.70274

**Published:** 2025-11-05

**Authors:** Yixin Ma, Hansol Lee, Kwok‐Shing Chan, Laleh Eskandarian, Kyla Gaudet, Qiyuan Tian, Aneri Bhatt, Julianna Gerold, Andrew W. Russo, David H. Salat, Eric C. Klawiter, Susie Y. Huang, Hong‐Hsi Lee

**Affiliations:** ^1^ Athinoula A. Martinos Center for Biomedical Imaging, Department of Radiology Massachusetts General Hospital Charlestown Massachusetts USA; ^2^ Department of Biomedical Engineering Ulsan National Institute of Science and Technology Ulsan South Korea; ^3^ Department of Neurology Massachusetts General Hospital, Harvard Medical School Boston Massachusetts USA; ^4^ Harvard‐MIT Division of Health Sciences and Technology Massachusetts Institute of Technology Cambridge Massachusetts USA

**Keywords:** aging, diffusion MRI, hippocampal microstructure, HippUnfold, SANDI model, super‐resolution

## Abstract

The hippocampus, a brain region critical for memory, undergoes significant age‐related changes at both the macroscopic and microstructural levels. This study investigates these changes using high‐gradient diffusion MRI (dMRI) data analyzed in an unfolded hippocampal space. We applied the Soma and Neurite Density Imaging (SANDI) model to quantify microstructural alterations in 72 cognitively healthy participants aged 19–85 years, scanned on a 3 T Connectome MRI scanner with a maximum gradient strength of 300 mT/m. By combining SANDI with a super‐resolution algorithm and the HippUnfold toolbox, we achieved high spatial fidelity in our analysis. We observed significant age‐related reductions in soma fraction and soma radius, particularly in the subiculum and dentate gyrus, alongside increases in extracellular diffusivity and extracellular fraction, indicating a decline in cellular density and structural integrity. These microstructural changes occur alongside macroscopic alterations such as reduced hippocampal volume and cortical thickness, decreased gyrification, and increased curvature in specific subfields. The spatial correlations between microstructural and macroscopic metrics across the unfolded hippocampal space are weak, both in their mean values and in how they change with age. Our findings suggest that SANDI metrics provide sensitive and complementary information to traditional structural measures, offering new insights into the microstructural underpinnings of hippocampal aging. This study highlights the potential of advanced dMRI techniques to detect subtle age‐related changes in hippocampal microstructure, which may contribute to our understanding of aging and its impact on memory and cognition.

## Graphical abstract

Using high‐gradient diffusion MRI with the SANDI model and HippUnfold for fine‐scale analysis, we detected localized hippocampal aging changes beyond volumetric/thickness reductions. Subiculum and dentate gyrus exhibit significantly reduced soma density/size and increased extracellular space, highlighting subtle microstructural alterations critical for a deeper understanding of hippocampal aging.

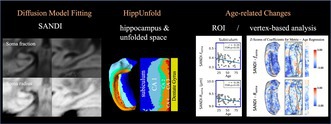

**Abbreviations**
AD: axial diffusivity
AK: axial kurtosis
CA: Cornu Ammonis
CA1, CA2, CA3, CA4: subfields of the Cornu Ammonis of the hippocampus
De: extracellular diffusivity
DG: dentate gyrus
Din: intracellular diffusivity
DKI: diffusion kurtosis imaging
DTI: diffusion tensor imaging
DWIs: diffusion‐weighted images
FA: fractional anisotropy
FDR: false discovery rate
fextra: extracellular fraction
ficvf: intra‐cellular volume fraction
fiso: fraction of isotropic water
fneurite: neurite fraction
fsoma: soma fraction
MD: mean diffusivity
MK: mean kurtosis
MRI: magnetic resonance imaging
NODDI: neurite orientation dispersion and density imaging
ODI: orientation dispersion index
RK: radial kurtosis
ROI: region of interest
Rsoma: soma radius
SANDI: soma and neurite density imaging
SNR: signal‐to‐noise ratio
T1_w_/T2_w_
: ratio of T1‐weighted images to T2‐weighted images
TE: echo time
TR: repetition time

## 1 Introduction

The hippocampus, essential to memory function, is particularly susceptible to age‐related changes, which manifest at both the macroscopic and microscopic levels (Bettio et al. 2017; Driscoll et al. 2003; O'Shea et al. 2016; Singh et al. 2024; Tanila et al. 1997). Structural magnetic resonance imaging (MRI) shows age‐related reductions in hippocampal volume (Adler et al. 2018; Fraser et al. 2021; Pereira et al. 2014; Raz et al. 2015; Shing et al. 2011). At the microscopic level, histological studies show cell body shrinkage, cell density reduction, and dendrite alterations with normal aging in pyramidal neurons of the subiculum and CA regions as well as granule and mossy cells of the dentate gyrus (Harding 1998; Ball 1977; Mani et al. 1986; Šimić et al. 1997; West 1993; West et al. 1994). Table 1 summarizes the cell types, average cell body diameters (in μm) (Benavides‐Piccione et al. 2019; Grovola et al. 2020; Ito 2014; López‐Meraz and Álvarez‐Croda 2023; Nadler and Zhan 2017; Spencer and Bland 2007), neuron counts (in millions) (Harding 1998; West et al. 1994), and observed neuron/dendrite/glial cell changes with aging (Boldrini et al. 2018; Ciric et al. 2019; Dickstein et al. 2007; Matias et al. 2021; Schneider et al. 2022; Su et al. 2022; West 1993) in the Subiculum, CA1, CA2–3, and DG regions of the hippocampus from previous literature. The average cell body diameters and neuron counts vary across subregions, reflecting both their unique roles and vulnerabilities in aging. These regional differences and the complexity of the involved cellular processes underscore the necessity for advanced imaging techniques capable of capturing these microstructural alterations in vivo.

**TABLE 1:** Summary of cellular characteristics and age‐related changes in hippocampal subregions. This table presents the cell types, average soma radius (in μm), neuron counts (in millions), neuron cell counting (in millions), and density (in 10^3^/mm^3^) and changes with aging across subfields of the hippocampus. Details in calculations are described in the Supporting Information.

Hippocampal subregions	Cell type	Average cell radius (μm)	Counting (millions)/density (10^3^/mm^3^)	Changes with aging
Subiculum	Pyramidal neurons	~7.8 μm (Rosenblum et al. 2024)	4.60/8.69 (Harding 1998) 5.95/NA (West et al. 1994)	Significant reduction in the neuronal density with aging (West et al. 1994)
CA1		7.5 ~ 10 μm (Benavides‐Piccione et al. 2019; Spencer and Bland 2007)	6.14/9.58 (Harding 1998) 14.08/NA (West et al. 1994)	Non‐significant neuronal density reduction with aging. While significant reduction in AD (West et al. 1994)
CA2‐3		~12.5 μm (Spencer and Bland 2007) *CA2‐3′s pyramidal soma size is larger than that of CA1 (Spencer and Bland 2007)	2.28/16.52 (Harding 1998) 2.83/NA (West et al. 1994)	Neuronal density does not significantly change with aging (West et al. 1994)
Dentate gyrus	Granule cells	2.5 ~ 4 μm (Ito 2014) 4 ~ 7.5 μm (Nadler and Zhan 2017; Spencer and Bland 2007)	11.25/225 (Harding 1998) 18.66/NA (granule cell layer) (West et al. 1994)	Neuronal density in hilus of dentate gyurs significantly decreases with aging (West 1993; West et al. 1994)
	Mossy cells	12 ~ 14 μm (pig) (Grovola et al. 2020)		
All regions	Glial cells	Astrocytes: 3.8 ~ 8.3 μm (Bedner et al. 2019) Microglia (rat): 1.3 ~ 2.7 μm (ramified), 2.5 ~ 5 μm (hypertrophic), 4.2 ~ 7.6 μm (bushy), 6.1 ~ 8.3 μm (amoeboid) (López‐Meraz and Álvarez‐Croda 2023)	Glial cell to neuron cell ratio = 51:47 (Su et al. 2022)	Astrocyte senescence (Matias et al. 2021) Microglial dystrophy (Streit et al. 2004)

**TABLE 2:** Summary of statistical results for structural, SANDI, NODDI, and DKI metrics across brain regions (Subiculum, CA1, CA2, CA3, CA4, and DG). The table displays the correlation coefficient (*r*) between each metric and age, as well as the *t*‐score and FDR‐adjusted *p*‐value from a *t*‐test on the age regression coefficient (β₁) in the model: Metric = β₀ + β₁ × Age + β₂ × Biological Sex + ε.

Metric	Subiculum	CA1	CA2	CA3	CA4	DG
Volume	*r* = −0.61 *t* = −6.06 FDR‐*p* = 1.92e‐06	*r* = −0.42 *t* = −3.74 FDR‐*p* = 1.77e‐03	*r* = −0.02 *t* = −0.14 FDR‐*p* = 9.72e‐01	*r* = −0.21 *t* = −1.79 FDR‐*p* = 1.38e‐01	*r* = −0.29 *t* = −2.41 FDR‐*p* = 3.98e‐02	*r* = −0.49 *t* = −4.62 FDR‐*p* = 1.39e‐04
Thickness	*r* = −0.53 *t* = −5.16 FDR‐*p* = 2.63e‐05	*r* = −0.39 *t* = −3.54 FDR‐*p* = 2.79e‐03	*r* = −0.11 *t* = −0.91 FDR‐*p* = 4.94e‐01	*r* = 0.02 *t* = 0.13 FDR‐*p* = 8.95e‐01	*r* = −0.10 *t* = −0.85 FDR‐*p* = 5.07e‐01	/
Gyrification	*r* = −0.46 *t* = −4.09 FDR‐*p* = 7.29e‐04	*r* = −0.34 *t* = −2.99 FDR‐*p* = 1.01e‐02	*r* = 0.04 *t* = 0.29 FDR‐*p* = 9.32e‐01	*r* = −0.27 *t* = −2.30 FDR‐*p* = 4.74e‐02	*r* = −0.29 *t* = −2.51 FDR‐*p* = 3.32e‐02	*r* = −0.36 *t* = −3.17 FDR‐*p* = 7.50e‐03
Curvature	*r* = 0.16 *t* = 1.31 FDR‐*p* = 2.96e‐01	*r* = 0.02 *t* = 0.18 FDR‐*p* = 9.82e‐01	*r* = 0.13 *t* = 1.11 FDR‐*p* = 3.86e‐01	*r* = 0.35 *t* = 3.02 FDR‐*p* = 1.03e‐02	*r* = −0.19 *t* = −1.41 FDR‐*p* = 2.69e‐01	*r* = −0.02 *t* = −0.13 FDR‐*p* = 9.35e‐01
SANDI‐*f*_soma_	*r* = −0.34 *t* = −2.85 FDR‐*p* = 2.70e‐02	*r* = −0.31 *t* = −2.56 FDR‐*p* = 4.31e‐02	*r* = −0.00 *t* = −0.04 FDR‐*p* = 9.70e‐01	*r* = −0.08 *t* = −0.60 FDR‐*p* = 6.16e‐01	*r* = −0.36 *t* = −2.98 FDR‐*p* = 2.95e‐02	*r* = −0.39 *t* = −3.33 FDR‐*p* = 1.33e‐02
SANDI‐*R*_soma_	*r* = −0.36 *t* = −2.97 FDR‐*p* = 2.58e‐02	*r* = −0.26 *t* = −2.16 FDR‐*p* = 1.03e‐01	*r* = −0.14 *t* = −1.15 FDR‐*p* = 3.98e‐01	*r* = −0.16 *t* = −1.25 FDR‐*p* = 3.52e‐01	*r* = −0.27 *t* = −2.16 FDR‐*p* = 9.66e‐02	*r* = −0.21 *t* = −1.70 FDR‐*p* = 2.28e‐01
SANDI‐*f*_extra_	*r* = 0.47 *t* = 4.17 FDR‐*p* = 1.76e‐03	*r* = 0.21 *t* = 1.66 FDR‐*p* = 2.17e‐01	*r* = 0.08 *t* = 0.61 FDR‐*p* = 6.31e‐01	*r* = 0.21 *t* = 1.67 FDR‐*p* = 2.23e‐01	*r* = 0.34 *t* = 2.83 FDR‐*p* = 2.56e‐02	*r* = 0.48 *t* = 4.36 FDR‐*p* = 1.73e‐03
SANDI‐*D*_e_	*r* = 0.40 *t* = 3.41 FDR‐*p* = 1.38e‐02	*r* = 0.18 *t* = 1.41 FDR‐*p* = 2.96e‐01	*r* = −0.02 *t* = −0.16 FDR‐*p* = 8.96e‐01	*r* = 0.03 *t* = 0.27 FDR‐*p* = 8.32e‐01	*r* = 0.34 *t* = 2.82 FDR‐*p* = 2.36e‐02	*r* = 0.34 *t* = 2.86 FDR‐*p* = 2.90e‐02
SANDI‐*f*_neurite_	*r* = 0.09 *t* = 0.67 FDR‐*p* = 6.26e‐01	*r* = 0.19 *t* = 1.51 FDR‐*p* = 2.71e‐01	*r* = −0.11 *t* = −0.85 FDR‐*p* = 5.52e‐01	*r* = −0.10 *t* = −0.77 FDR‐*p* = 5.96e‐01	*r* = −0.09 *t* = −0.67 FDR‐*p* = 6.48e‐01	*r* = −0.05 *t* = −0.36 FDR‐*p* = 7.86e‐01
SANDI‐*D*_in_	*r* = 0.09 *t* = 0.67 FDR‐*p* = 6.07e‐01	*r* = −0.15 *t* = −1.15 FDR‐*p* = 3.83e‐01	*r* = −0.24 *t* = −1.91 FDR‐*p* = 1.58e‐01	*r* = −0.18 *t* = −1.42 FDR‐*p* = 3.04e‐01	*r* = 0.12 *t* = 0.97 FDR‐*p* = 4.86e‐01	*r* = 0.18 *t* = 1.40 FDR‐*p* = 2.85e‐01
NODDI‐*f*_icvf_	*r* = 0.30 *t* = 2.60 FDR‐*p* = 5.75e‐02	*r* = 0.20 *t* = 1.63 FDR‐*p* = 2.55e‐01	*r* = 0.21 *t* = 1.78 FDR‐*p* = 2.20e‐01	*r* = 0.09 *t* = 0.72 FDR‐*p* = 6.92e‐01	*r* = 0.11 *t* = 0.88 FDR‐*p* = 6.33e‐01	*r* = 0.19 *t* = 1.51 FDR‐*p* = 2.97e‐01
NODDI‐*f*_iso_	*r* = 0.51 *t* = 4.83 FDR‐*p* = 2.89e‐04	*r* = 0.18 *t* = 1.46 FDR‐*p* = 3.05e‐01	*r* = 0.04 *t* = 0.33 FDR‐*p* = 8.91e‐01	*r* = 0.05 *t* = 0.40 FDR‐*p* = 8.57e‐01	*r* = 0.31 *t* = 2.66 FDR‐*p* = 5.94e‐02	*r* = 0.41 *t* = 3.63 FDR‐*p* = 5.27e‐03
NODDI‐ODI	*r* = 0.00 *t* = 0.01 FDR‐*p* = 9.94e‐01	*r* = 0.54 *t* = 5.30 FDR‐*p* = 9.12e‐05	*r* = 0.21 *t* = 1.69 FDR‐*p* = 2.36e‐01	*r* = 0.19 *t* = 1.55 FDR‐*p* = 2.89e‐01	*r* = 0.02 *t* = 0.16 FDR‐*p* = 9.15e‐01	*r* = 0.18 *t* = 1.50 FDR‐*p* = 2.97e‐01
DKI‐AD	*r* = 0.38 *t* = 3.32 FDR‐*p* = 1.23e‐02	*r* = −0.17 *t* = −1.41 FDR‐*p* = 3.25e‐01	*r* = −0.10 *t* = −0.82 FDR‐*p* = 6.50e‐01	*r* = −0.06 *t* = −0.48 FDR‐*p* = 8.23e‐01	*r* = 0.22 *t* = 1.82 FDR‐*p* = 2.10e‐01	*r* = 0.23 *t* = 1.90 FDR‐*p* = 1.84e‐01
DKI‐RD	*r* = 0.35 *t* = 3.00 FDR‐*p* = 2.82e‐02	*r* = −0.03 *t* = −0.26 FDR‐*p* = 9.07e‐01	*r* = −0.03 *t* = −0.25 FDR‐*p* = 9.00e‐01	*r* = 0.03 *t* = 0.25 FDR‐*p* = 8.87e‐01	*r* = 0.28 *t* = 2.41 FDR‐*p* = 8.14e‐02	*r* = 0.32 *t* = 2.82 FDR‐*p* = 4.14e‐02
DKI‐MD	*r* = 0.47 *t* = 4.23 FDR‐*p* = 1.26e‐03	*r* = −0.10 *t* = −0.80 FDR‐*p* = 6.53e‐01	*r* = −0.06 *t* = −0.50 FDR‐*p* = 8.30e‐01	*r* = −0.01 *t* = −0.08 FDR‐*p* = 9.50e‐01	*r* = 0.30 *t* = 2.56 FDR‐*p* = 6.00e‐02	*r* = 0.25 *t* = 2.14 FDR‐*p* = 1.33e‐01
DKI‐AK	*r* = −0.02 *t* = −0.19 FDR‐*p* = 9.16e‐01	*r* = 0.48 *t* = 4.52 FDR‐*p* = 5.73e‐04	*r* = 0.20 *t* = 1.69 FDR‐*p* = 2.41e‐01	*r* = 0.07 *t* = 0.54 FDR‐*p* = 8.16e‐01	*r* = 0.04 *t* = 0.32 FDR‐*p* = 8.82e‐01	*r* = 0.24 *t* = 2.03 FDR‐*p* = 1.44e‐01
DKI‐RK	*r* = 0.15 *t* = 1.21 FDR‐*p* = 4.23e‐01	*r* = 0.11 *t* = 0.91 FDR‐*p* = 6.23e‐01	*r* = −0.11 *t* = −0.86 FDR‐*p* = 6.32e‐01	*r* = −0.25 *t* = −2.13 FDR‐*p* = 1.28e‐01	*r* = 0.02 *t* = 0.17 FDR‐*p* = 9.19e‐01	*r* = 0.04 *t* = 0.33 FDR‐*p* = 9.05e‐01
DKI‐MK	*r* = 0.14 *t* = 1.12 FDR‐*p* = 4.76e‐01	*r* = 0.28 *t* = 2.36 FDR‐*p* = 8.84e‐02	*r* = 0.02 *t* = 0.14 FDR‐*p* = 9.18e‐01	*r* = −0.08 *t* = −0.61 FDR‐*p* = 7.62e‐01	*r* = 0.03 *t* = 0.27 FDR‐*p* = 9.16e‐01	*r* = 0.17 *t* = 1.36 FDR‐*p* = 3.47e‐01
DKI‐FA	*r* = 0.09 *t* = 0.74 FDR‐*p* = 6.91e‐01	*r* = −0.31 *t* = −2.61 FDR‐*p* = 6.13e‐02	*r* = −0.15 *t* = −1.23 FDR‐*p* = 4.23e‐01	*r* = −0.13 *t* = −1.09 FDR‐*p* = 4.87e‐01	*r* = −0.08 *t* = −0.70 FDR‐*p* = 7.02e‐01	*r* = −0.05 *t* = −0.42 FDR‐*p* = 8.55e‐01
T1_w_/T2_w_	*r* = 0.48 *t* = 4.16 FDR‐*p* = 1.40e‐03	*r* = 0.45 *t* = 3.92 FDR‐*p* = 2.53e‐03	*r* = 0.06 *t* = 0.49 FDR‐*p* = 8.22e‐01	*r* = 0.22 *t* = 1.73 FDR‐*p* = 2.35e‐01	*r* = 0.28 *t* = 2.26 FDR‐*p* = 1.05e‐01	*r* = 0.26 *t* = 2.04 FDR‐*p* = 1.51e‐01

Advancements in diffusion MRI (dMRI) allow probing hippocampal microstructure non‐invasively. Diffusion tensor imaging (DTI) models diffusion using an ellipsoid tensor, revealing anisotropy and mean diffusivity linked to cellular density and fiber organization (Le Bihan et al. 2001). Studies show age‐related decreases in fractional anisotropy (FA) and increases in mean diffusivity (MD) in the hippocampus (Haller et al. 2019; Pereira et al. 2014; Solar et al. 2021). Neurite Orientation Dispersion and Density Imaging (NODDI), separating intra‐neurite, extracellular, and free‐water diffusion (Zhang et al. 2012), has revealed age‐related increases in restricted, hindered, and free diffusion (Radhakrishnan et al. 2020; Venkatesh et al. 2020), likely induced by cell density reduction, loss of cell integrity, and increase in extracellular space (Raz and Rodrigue 2006; Syková and Nicholson 2008).

Recent advances in dMRI models for the brain gray matter offer important advantages over the conventional diffusion models, particularly for the hippocampus, a gray matter structure composed of densely packed neuronal cell bodies and dendrites (Andersen et al. 2006; Duvernoy et al. 2005). For example, the Soma and Neurite Density Imaging (SANDI) model quantifies tissue metrics of soma, neurite, and extracellular space separately (Palombo et al. 2020). We propose that SANDI's soma radius and fraction may serve as in vivo markers of neuron cell body shrinkage and reduced density due to aging, while the neurite fraction could capture dendritic alterations. SANDI has been successfully applied to examine microstructural alterations in various neurological conditions, including multiple sclerosis (Krijnen et al. 2023; Margoni et al. 2023), and to investigate cortical gray matter alteration associated with aging (Lee, Lee, et al. 2024; Singh et al. 2024) and brain development (Genc et al. 2025; Karat et al. 2024). Thus, applying SANDI to the hippocampus could yield valuable insights into age‐related microstructural alterations in the soma and neuronal compartments.

In this study, we focus on investigating subtle, region‐specific age‐related alterations within the hippocampus, a small volume of 3–3.5 cm^3^ (Suzuki et al. 2005) with its complex, folded architecture (Pluta 2021; Swanson 1979). Although direct high‐resolution (≤ 1 mm) DWI acquisition could theoretically provide the necessary details in the hippocampus, it is often impractical due to low SNR and lengthy scan times. To overcome these challenges, we apply super‐resolution algorithms to reconstruct high‐resolution data from scans of the lower‐resolution dMRI data (Coupé et al. 2013; Lee et al. 2019; Manjón et al. 2010). This algorithm enhances the spatial fidelity of diffusion‐weighted images (DWIs) by leveraging self‐similarity, wherein high spatial frequency details from high‐resolution anatomical images are effectively transferred to low‐resolution DWIs, significantly reducing partial volume effects in the derived diffusion metrics.

Advances in automatic hippocampal segmentation tools, such as the HippUnfold framework (DeKraker et al. 2021, 2022), improve delineation of the hippocampus and its substructures. It incorporates advanced unfolding techniques that generate geodesic coordinate frameworks. This allows unwrapping 3D hippocampal volume onto 2D surface spaces for more accessible and detailed examination of spatial patterns in the unfolded hippocampal space (DeKraker et al. 2021, 2022). The HippUnfold toolbox enables morphological measurements such as mean curvature, gyrification, and thickness. It has been successfully applied to study hippocampal perfusion properties (Haast et al. 2023), diffusion‐microstructural metrics in the developing brain (Karat et al. 2024), and hippocampal geometry (Diers et al. 2023), with each study revealing characteristic patterns across hippocampal subfields. We propose combining super‐resolution and HippUnfold to identify microstructural alterations in smaller regions than previously detectable, thereby enhancing our understanding of hippocampal aging.

Leveraging SANDI diffusion modeling, super‐resolution, and the HippUnfold segmentation and surface unwrapping, this study aims to investigate localized microstructural variations and their alterations with age. Our analysis investigates age‐associated changes in both macroscopic metrics—including hippocampal volume, cortical thickness, gyrification, and curvature—and microstructural metrics such as soma radius, soma density fractions, and extracellular diffusivity. Additionally, we assess the relationships between these macroscopic and microstructural metrics. We analyze diffusion MRI data from 72 participants ranging in age from 19 to 85 years on the Connectome MRI system with a maximum gradient strength of 300 mT/m. We evaluate the regional variations in hippocampal microstructural parameters with respect to age, with the broader goal of advancing a greater understanding of the mechanisms associated with normal aging.

## 2 Results

We first present the changes in macroscopic and microstructural metrics with normal aging at the hippocampal subfield level, with statistical results summarized in Table 2. Following this, we proceed to an examination of these metrics at higher spatial resolution, leveraging the vertices on the hippocampal mid‐thickness surfaces to display both the mean values and age‐related correlations of these features, aiming to understand not only the overall trends within hippocampal subfields but also the localized variations and specific vertices where significant changes occur.

### 2.1 Age‐Dependent Changes in Metrics at the Hippocampal Subfield Level

#### 2.1.1 Macroscopic Metrics

**FIGURE 1:**
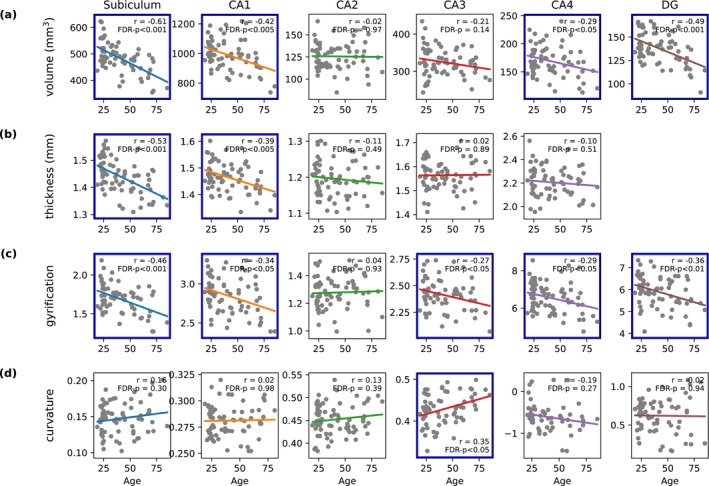
Age‐related changes in structural metrics of hippocampal subfields. Correlation between age and structural metrics (volume, thickness, gyrification, and curvature) in hippocampal subfields (Subiculum, CA1, CA2, CA3, CA4, DG). Each scatter plot displays the correlation coefficient (*r*) and *p*‐value. Significant correlations after FDR correction are marked with blue boxes.

Figure 1 demonstrates how the macroscopic metrics of the hippocampal subfields vary with age. Volume and thickness metrics both showed a clear decline with age in the subiculum and CA1, with the subiculum showing the most significant age‐related alteration (volume: *r* = −0.61, FDR *p* < 0.001; thickness: *r* = −0.53, FDR *p* < 0.001). Gyrification showed a significant decrease with age in all subfields except for CA2. Curvature only showed a significant increase in CA3 with age (*r* = 0.35, FDR *p* < 0.05).

#### 2.1.2 SANDI Metrics

**FIGURE 2:**
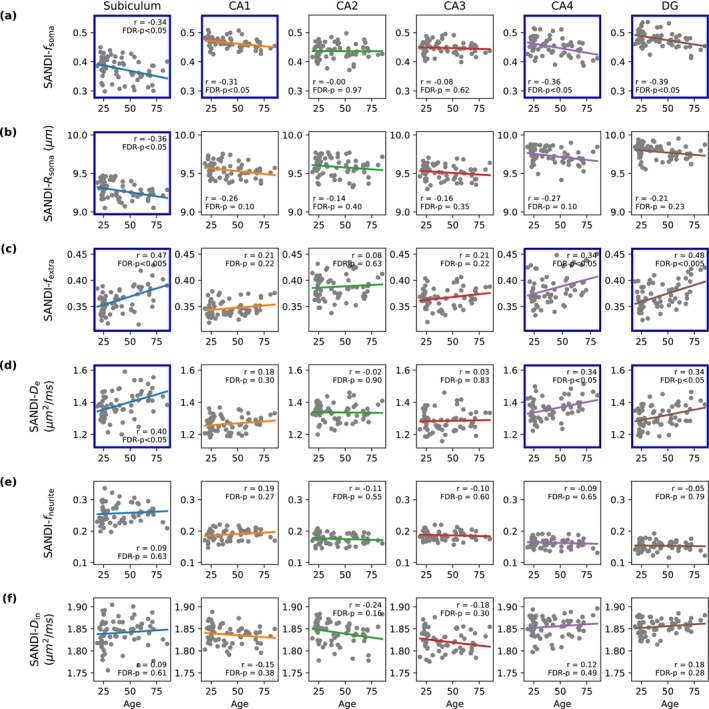
Age‐related changes in SANDI metrics of hippocampal subfields. Correlation between age and SANDI metrics (soma fraction *f*_soma_, soma radius *R*_soma_, extracellular fraction *f*_extra_, extracellular diffusivity *D*_extra_, neurite fraction *f*_neurite_, and intracellular diffusivity *D*_in_) across hippocampal subfields. Each scatter plot shows the correlation coefficient (*r*) and *p*‐value, with significant results highlighted in blue boxes after FDR correction.

Figure 2 shows how the SANDI metrics vary with age in the hippocampus. The soma signal fraction (*f*_soma_) and soma radius (*R*_soma_) significantly decreased with age in the subiculum (*f*_soma_: *r* = −0.34, FDR *p* < 0.05; *R*_soma_: *r* = −0.36, FDR *p* < 0.05). *f*_soma_ also significantly decreased with aging in CA1 (*f*_soma_: *r* = −0.31, FDR *p* < 0.05), in CA4 (*f*_soma_: *r* = −0.36, FDR *p* < 0.05), and in dentate gyrus (DG) (*f*_soma_: *r* = −0.39, FDR *p* < 0.005). Furthermore, the fraction of extracellular space (*f*_extra_) increased with age in the subiculum (*f*_extra_: *r* = 0.47, FDR *p* < 0.005), CA4 (*f*_extra_: *r* = 0.34, FDR *p* < 0.05), and DG (*f*_extra_: *r* = 0.48, FDR *p* < 0.005). The extracellular diffusivity (*D*_e_) increased significantly with age in the subiculum (*D*_e_: *r* = 0.40, FDR *p* < 0.05), CA4 (*D*_e_: *r* = 0.34, FDR *p* < 0.05), and DG (*D*_e_: *r* = 0.34, FDR *p* < 0.05). On the other hand, the *f*_neurite_ and *D*_in_ showed no significant alterations with age across all subfields.

#### 2.1.3 NODDI, DKI Metrics, and T1_w_/T2_w_ Ratio

To place the results of our advanced dMRI results with SANDI in the context of existing dMRI metrics such as NODDI and diffusion kurtosis imaging (DKI) (Jensen et al. 2005), Figure S1 presents the age‐related changes in NODDI and DKI metrics across hippocampal subfields. The NODDI metrics showed significant increases with age in the fraction of isotropic diffusion (*f*_iso_) in the subiculum (*r* = 0.51, FDR *p* < 0.001) and DG (*r* = 0.41, FDR *p* < 0.01). Orientation dispersion index (ODI) increased significantly in CA1 (*r* = 0.54, FDR *p* < 0.001). The DKI metrics, particularly axial diffusivity (AD), showed significant increases in the subiculum (AD: *r* = 0.38, FDR *p* < 0.05). Mean kurtosis (MK), axial kurtosis (AK), and radial kurtosis (RK) exhibited complex trends, only showing significant increases of AK in CA1 (*r* = 0.48, FDR *p* < 0.001). The T1_w_/T2_w_ ratio, used as a surrogate imaging marker for myelin content in the brain (Glasser and Van Essen 2011; Lee, Woo, et al. 2024), increased significantly with age in the subiculum (*r* = 0.48, FDR *p* < 0.005) and CA1 (*r* = 0.45, FDR *p* < 0.005).

### 2.2 Spatial Distribution of Mean Metrics on the Hippocampal Surface Averaged Over All Subjects (Ages 19–85)

#### 2.2.1 Macroscopic Metrics

**FIGURE 3:**
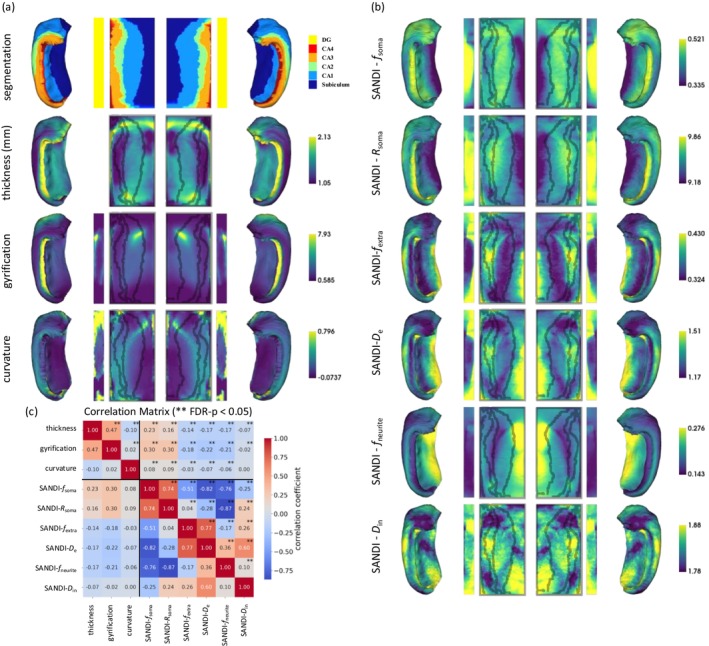
Mean values and spatial distribution of structural and SANDI metrics. (a) Mean values of structural metrics (thickness, gyrification, curvature) across all subjects displayed on the unfolded hippocampal surface. (b) Mean SANDI metrics shown on the same surface. (c) Correlation matrix showing the spatial relationships among structural and SANDI metrics.

Figure 3a presents the mean values of the macrostructural metrics averaged over all ages across the hippocampal unfolded and folded surfaces. Our mean structural metric maps averaged over subjects aged 19–85 exhibit spatial distribution patterns consistent with those observed from previous studies of HippUnfold applied to the developing brain aged 22–35 (Karat et al. 2023). We observed that thickness was highest in the anterior and posterior poles of the subiculum and CA4 subfields, was intermediate in the body of CA1 and subiculum, and was lowest in the body of CA2/CA3. The subiculum and CA1 subfields (located around 20% along the long A‐*P* axis) exhibited higher gyrification values. Lower gyrification was shown in the CA2 and CA3 subfields. Higher curvature values were observed in the dentate gyrus, indicating more pronounced surface bending in the dentate gyrus. Lower curvature values were observed in the body‐tail of the subiculum and CA1 regions.

#### 2.2.2 SANDI Metrics

*f*_soma_ was the highest in the body of the DG, indicating a higher density in this region, consistent with neuron counts of 11.25–18.66 million, and highest neuron density 225 (10^3^/mm^3^) in DG (Harding 1998; West et al. 1994), as shown in Table 1 from histology literature reviews. Conversely, *f*_soma_ was low in the body of the subiculum, suggesting a lower density, which aligns with neuron counts of 4.6–5.95 million, and lowest neuron density 8.69 (10^3^/mm^3^) in subiculum (Harding 1998; West et al. 1994), also shown in Table 1. The spatial distribution of *f*_soma_ and *R*_soma_ differed in the CA regions: When comparing CA1 to CA2/3, *f*_soma_ was higher while *R*_soma_ was moderately lower in CA1. These findings are consistent with histological observations of more densely packed pyramidal neurons in CA1 that have smaller cell bodies compared to those in CA2/3 (Dudek et al. 2016). These findings correspond with the quantitative histology data in Table 1, where CA1 pyramidal neurons have an average diameter of 15–20 μm, while those in CA2/3 are around 25 μm in diameter (Spencer and Bland 2007).

The extracellular diffusivity (*D*_e_) and extracellular fraction (*f*_extra_) were higher along the body‐tail of the subiculum and at the anterior and posterior poles of the DG. Lower *D*_e_ was observed in the body of the DG and the anterior segments of CA1‐3.

The intracellular diffusivity (*D*_in_) was higher at the anterior and posterior poles of the DG, in the mid‐body of CA2‐3, and in the lower tail of the subiculum. Neurite fraction (*f*_neurite_) was highest in the body of the subiculum.

#### 2.2.3 Spatial Correlation Between Structural and SANDI Metrics

The correlation matrix in Figure 3c demonstrates the spatial relationships among the various structural and SANDI metrics. The analysis reveals a weak correlation between structural metrics and SANDI metrics (all |*r*| ≤ 0.3).

Among the macroscopic metrics: Hippocampal thickness positively correlated with gyrification (*r* = 0.47) and showed a negligible correlation with curvature (*r* = 0.10). The correlation between curvature and gyrification was low (*r* = 0.02).

Among the SANDI metrics: *D*_e_ was positively correlated with *f*_extra_ (*r* = 0.77), suggesting a relationship between extracellular diffusivity and extracellular fraction. *f*_soma_ positively correlated with *R*_soma_ (*r* = 0.74), whereas *f*_*s*oma_ negatively correlated with *D*_e_ (*r* = −0.82) and *f*_neurite_ (*r* = −0.76).

#### 2.2.4 DKI, NODDI, and T1_w_/T2_w_ Ratio

As shown in Figure S2, AD and RD shared similar spatial distributions with higher values in the anterior and posterior poles of the DG and in CA2 at 40%–50% of the long [anterior–posterior (AP)] axis. However, AD and RD slightly differed in spatial distribution in the subiculum: AD showed high diffusivity in the subiculum from 40 to 100% along the AP long axis, while RD peaked in the subiculum from 80 to 100% along the AP long axis.

AK was the highest near the subiculum–CA1 interface. RK was the highest in the body of the subiculum. FA and the T1_w_/T2_w_ ratio both peaked in the body of the subiculum.

For NODDI, the intra‐cellular volume fraction (*f*_icvf_) was the highest in the body of the subiculum, while the isotropic volume fraction (*f*_iso_) reached its peak in the subiculum at 40%–100% along the AP long axis. ODI was higher in CA1 compared to CA2/3.

The correlation matrix in Figure S2 shows that, among the DKI metrics, there was a strong positive correlation between AD, RD, and MD, indicating that, in the hippocampus, regions with high diffusivity in one direction tended to exhibit similar properties in the orthogonal direction. T1_w_/T2_w_ ratio was positively correlated with FA (*r* = 0.76) and with NODDI‐ *f*_icvf_ (*r* = 0.80). In addition, NODDI‐ODI was negatively correlated with FA (*r* = −0.85); AD was positively correlated with NODDI‐ *f*_iso_ (*r* = 0.77); and AD was negatively correlated with NODDI‐ODI (*r* = −0.74).

### 2.3 Spatial Distribution of Age‐Dependent Changes in Metrics on Unfolded Hippocampal Surface

**FIGURE 4:**
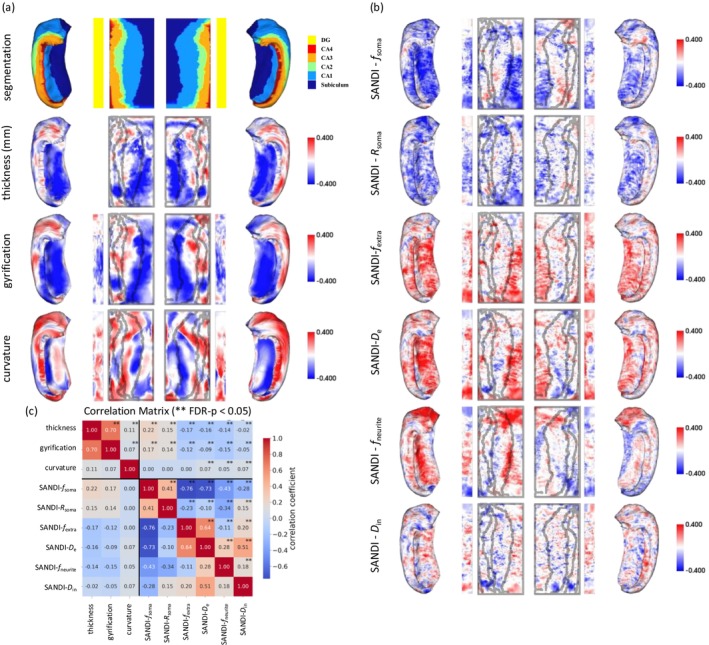
Age‐related changes in structural and SANDI Metrics on the unfolded hippocampal surface. Correlation coefficients between age and each metric [structural in (a) and SANDI in (b)] are shown on the unfolded hippocampal surface. Positive and negative correlations are color‐coded. The correlation matrix in (c) shows the spatial relationships among structural and SANDI metrics.

Figure 4 and Figure S3 illustrate the correlation coefficients between age and macroscopic and microscopic metrics mapped onto the unfolded hippocampal surfaces, with positive correlations color‐coded in blue and negative correlations color‐coded in red. Building upon the subfield‐level changes presented in Figures 1 and 2 and Figure S1, these detailed surface maps offer a more fine‐grained spatial resolution of age‐dependent alterations, allowing us to observe regional variations within the subfields more precisely.

#### 2.3.1 Macroscopic Metrics

We observed a negative correlation between cortical thickness and age, particularly in the middle‐posterior portion of the subiculum and proximal CA1, indicating an age‐related decline in cortical thickness. Gyrification and age showed negative correlations mainly in the subiculum and DG. The correlations between curvature and age showed complicated regional variations with no clear trend.

#### 2.3.2 SANDI Metrics

Both *f*_soma_ and *R*_soma_ were negatively correlated with age, particularly in the body of DG, the body‐posterior regions of the subiculum, and the anterior pole of the CA regions. (Castro et al. 2022; Šimić et al. 1997; West 1993).

Both *D*_e_ and *f*_extra_ exhibited positive correlations with age, mainly in the subiculum at 40–100% along the AP long axis, and in the anterior and posterior poles of the DG.

The correlation between *D*_in_ and age did not show a significant regional pattern, whereas *f*_neurite_ increased with age in the anterior pole of the hippocampus.

#### 2.3.3 DKI, NODDI, and T1_w_/T2_w_ Ratio

Figure S3 shows that the diffusivities (AD, RD, and MD), NODDI‐*f*_icvf_, and *f*_iso_ increased with age in the subiculum at 40%–100% along the AP long axis, and in the anterior and posterior poles of DG. AK increased with age in CA1 and decreased with age in the tail of subiculum. T1_w_/T2_w_ ratio increased with age in the subiculum and CA1 and the body of the DG. In the subiculum at 50%–100% along the AP long axis, NODDI‐ODI decreased, and FA increased; in CA1, NODDI‐ODI increased and FA decreased.

## 3 Discussion

This study provides new insights into the complex patterns of age‐related structural and microstructural changes within the hippocampus, leveraging advanced diffusion MRI techniques and novel gray matter microstructural metrics such as those derived from the SANDI model. Our findings highlight significant decreases in volume and gyrification across most subfields and a distinct increase in curvature within the CA3 subfield, reflecting potential morphological adjustments with aging. Moreover, the application of SANDI enables detection of subtle microstructural alterations with age, including reductions in soma fraction and soma radius, alongside increases in extracellular diffusivity and fraction, particularly within the subiculum and dentate gyrus. These changes suggest a decline in cellular density and structural integrity, consistent with histological findings in aging brains. The observed relationships between macroscopic and microstructural metrics underscore the complementary nature of these measures, offering a more comprehensive understanding of hippocampal aging.

### 3.1 Age‐Related Alterations in Macroscopic and Diffusion‐Metrics Maps

#### 3.1.1 Age‐Related Alterations in Hippocampal Thickness and Volume

Our regression analysis of hippocampal volume versus age (Figure 1a) aligns with prior research showing age‐related hippocampal atrophy, particularly in the subiculum, CA1, CA4, and DG (Adler et al. 2018; Fraser et al. 2021; Pereira et al. 2014; Raz et al. 2015; Shing et al. 2011). Traditionally, hippocampal atrophy has been quantified using its absolute volume. Here, we utilized the thickness metrics provided by the HippUnfold tool to evaluate changes in hippocampal subregional structure with normal aging, analogous to a recently released study on HippUnfold applied to the developing brain (Karat et al. 2024). Notably, two macroscopic metrics, hippocampal volume and thickness measurements, are strongly correlated (Pearson‐*r* = 0.740), indicating that volume and thickness are both informative metrics to evaluate hippocampal atrophy.

#### 3.1.2 Age‐Related Alterations in Hippocampal Gyrification and Curvature

We used the gyrification and curvature metrics to evaluate the morphology of the hippocampal subfields in the context of normal aging, facilitated by the HippUnfold toolbox. When comparing and understanding these two metrics, it is important to note that gyrification is defined relative to the choice of unfolded space and does not distinguish between gyri and sulci. In contrast, curvature provides a more mathematical definition of a curved surface, with gyri and sulci having different signs. Our findings reveal a significant age‐related decrease in gyrification across most hippocampal subfields (Figure 1c), accompanied by a distinct age‐related increase in curvature, specifically in the CA3 subfield (Figure 1d). While no prior studies have focused on gyrification and curvature within the hippocampus during normal aging, existing research on cortical regions provides a relevant context. In the cortical gray matter, studies have documented a linear decrease in gyrification with age (Hogstrom et al. 2013; Lamballais et al. 2020); while gyrification generally decreases across subfields, curvature shows a more complex pattern—possibly because cortical thinning and atrophy associated with aging tend to lead to gyri becoming more sharply and steeply curved, while sulci tend to flatten and lose curvature. Furthermore, the observed increase in curvature within CA3 is the most pronounced among hippocampal subfields, on the order of what has been observed in cortical gyri (Lin et al. 2021; Magnotta et al. 1999).

#### 3.1.3 Age‐Related Alterations in SANDI Metrics

The application of SANDI to the hippocampus enables the detection of subtle microstructural alterations beyond the macroscopic changes with age. Our findings revealed significant age‐related reductions in both soma fraction (*f*_*s*oma_) and soma radius (*R*_soma_) across hippocampal subregions, particularly in the subiculum and DG (Figure 2a,b). These changes were accompanied by increases in extracellular fraction (*f*_extra_) and extracellular diffusivity (*D*_e_), suggesting a decline in cellular density and structural integrity with aging (Figure 2c,d). These findings are consistent with histological findings of neuron cell loss and cell body shrinkage that are most significant in the subiculum and DG (Castro et al. 2022; Šimić et al. 1997; West 1993). The observed increases in *f*_extra_ and *D*_e_ may reflect an expansion of the extracellular space due to cellular loss and shrinkage, which has been previously associated with cognitive decline in aging (Syková and Nicholson 2008). The relationship between SANDI metrics and the macroscopic measurements is particularly noteworthy (Figure 3c). While both macroscopic and microstructural changes were observed in the hippocampus with aging, the correlation matrix analysis revealed that the SANDI metrics provided information that was not contained in the thickness, gyrification, and curvature measures.

The age‐related changes in SANDI metrics (Figure 4) across the hippocampus revealed distinct regional patterns. We observed that the posterior subiculum exhibited the most significant age‐related alterations in SANDI metrics. The subiculum is a critical region that connects the fibers entering and leaving the hippocampus and plays a key role in mediating hippocampal‐cortical interactions (O'Mara et al. 2001). Based on our findings, the loss of cellular density and reduction in cellular size associated with the subiculum suggest that it is potentially the most sensitive region reflecting the loss of intra‐hippocampal and/or cortical‐hippocampal connections.

#### 3.1.4 Comparing Microscopic and Macroscopic Correlations With Age

Prior work (Lee, Lee, et al. 2024; Singh et al. 2024) reported that, in cortical gray matter, the correlations of alterations in tissue microstructure from dMRI and age were stronger than or comparable with those from macroscopic measures. In this study, we used the same dataset as in (Lee, Lee, et al. 2024) and showed that in the hippocampus, microstructural metrics from dMRI models and macroscopic metrics from anatomical MRI have comparable correlations with age, though macroscopic metrics have slightly stronger correlations with age than those of the microstructural metrics. However, we demonstrated that microstructural and macroscopic metrics provide independent information, that is, they are orthogonal to each other, highlighting the value of having both (Figures 3c and 4c). The complexity of the hippocampus potentially requires more information for evaluating the effect of aging.

#### 3.1.5 Comparison With Age‐Related Alterations in the Developing Brain

Our findings on age‐related microstructural alterations in the hippocampus with aging complement recent research on the developing hippocampus. Karat et al. evaluated SANDI metrics in the developing hippocampus in a cohort spanning the ages of 8–19 years (Karat et al. 2024) and found an increase in the neurite fraction and a decrease in extracellular fraction in the developing hippocampus, which is in line with the marked neural growth and pruning that occur in development. Our findings of reduced cell body size and density suggest that the aging hippocampus is primarily characterized by cellular shrinkage and loss. The findings of Karat et al. contrast with our results in the aging brain, underscoring the divergent nature of hippocampal microstructural alterations across the lifespan.

#### 3.1.6 Age‐Related Alterations in NODDI Metrics

Consistent with earlier hippocampal NODDI studies (Radhakrishnan et al. 2020; Venkatesh et al. 2020), we observed (1) significant increases of isotropic (freewater) fraction (NODDI‐*f*_*i*so_) with aging in the subiculum and dentate gyrus and (2) significant increases of orientation dispersion index (NODDI‐ODI) with aging in CA. Furthermore, SANDI provided additional interpretations at the cellular compartment level; we observed significant increases of extracellular fraction SANDI‐*f*_extra_ with aging, in parallel with increases of NODDI‐*f*_iso_ with aging.

#### 3.1.7 Age‐Related Alterations in DKI Metrics

In subiculum and dentate gyrus, we observed significant increases in radial diffusivity (DKI‐RD) with aging. Similarly, SANDI results showed significant increases in extracellular signal fraction (SANDI‐*f*_extra_) and the extracellular diffusivity (SANDI‐*D*_e_) with aging in the two regions, suggesting that the diffusivity increases with aging might be related to the enlarged, less hindered extracellular space.

#### 3.1.8 Correlation of DKI With NODDI and SANDI Metrics

##### 3.1.8.1 Model Assumptions

DKI is a signal representation with no specific interpretations of tissue microstructure, and it is challenging to interpret its findings with specific microstructural alterations. In contrast, biophysical models of multiple compartments enable one to distinguish signal contributions from each component (e.g., soma, neurite, extracellular space) (Coelho et al. 2019; Novikov et al. 2018; Palombo et al. 2020; Zhang et al. 2012), allowing more specific microstructural interpretations.

In this study, we choose to display NODDI results in Supporting Information for comparability with other previous literature (Radhakrishnan et al. 2020; Venkatesh et al. 2020), since NODDI is designed for microstructural imaging in white matter and may be less applicable to probing gray matter.

Instead, the SANDI model incorporates a soma compartment that is abundant in the gray matter and releases multiple assumptions used in NODDI. Furthermore, a previous study (Palombo et al. 2020) has shown that the exchange effect is negligible at short diffusion times < 20 ms, cf. diffusion time = 19 ms in this study. Therefore, SANDI is more suitable to apply in gray matter, and we showed SANDI results as the main findings.

##### 3.1.8.2 Sensitivity to Aging

In hippocampus (Figure 2 and Figure S1), we observed that (1) in subiculum and dentate gyrus, SANDI metrics showed significant age‐related alterations, similar to those of diffusivity from DKI (MD/AD/RD), (2) in CA1, SANDI‐*f*_soma_ showed significant age‐related alterations, though they are slightly lower than (but still comparable with) those of DKI‐AK and NODDI‐ODI, and (3) in CA4, SANDI metrics showed significant age‐related alterations, whereas DKI and NODDI metrics showed no significant results.

In other words, in subiculum and dentate gyrus, SANDI, NODDI, and DKI metrics showed comparable sensitivity to aging. In CA1, NODDI‐ODI and DKI‐AK showed slightly higher sensitivity to aging, compared with SANDI‐*f*_soma_. However, in CA4, SANDI uniquely showed high sensitivity to aging, which was not observed in NODDI and DKI results. All three models bring complements to each other as investigating the aging effect in hippocampus.

##### 3.1.8.3 Scan Time Comparison for Each Model

The number of acquired diffusion data for each model fitting was reported in our previous literature (Tian et al. 2022) and shortly summarized as follows. We fitted both DKI and NODDI models to diffusion data of four *b*‐values (*b* = 800, 1500, 2400, 3450 s/mm^2^ at short diffusion time = 19 ms) composed of 192 diffusion‐weighted images and 16 b0 images (≈13.5 min). We fitted the SANDI model to diffusion data of eight *b*‐values (*b* = 50, 350, 800, 1500, 2400, 3450, 4750, 6000 s/mm^2^ at short diffusion time = 19 ms) composed of 384 diffusion‐weighted images and 32 b0 images (≈27 min). In terms of minimal requirement, both DKI and NODDI require at least two *b*‐values, and SANDI requires at least five *b*‐values. To generate high‐quality parametric maps, we chose to use many *b*‐shells more than the minimal requirement of each model. The long diffusion protocol of multiple *b*‐shells offered cell‐type‐specific SANDI metrics—particularly soma fraction and radius—that provided biological‐specific insight unavailable from DKI and NODDI.

Further, to enhance the accessibility of the SANDI model on clinical scanners, the SANDI model can be further modified to work with diffusion data acquired using fewer *b*‐shells and lower *b*‐values within shorter scan times (Gyori et al. 2021). This can be achieved by applying the diffusion tortuosity relation in extracellular space, derived from effective medium theory for grains immersed in a matrix (Giordano 2003; Latour et al. 1994). However, this is out of the scope of the study and will be reported in the future.

##### 3.1.8.4 Applicability on Other High‐Gradient Performance Systems

Compared with DKI and NODDI, the SANDI model required more *b*‐shells at higher *b*‐values, which was previously only achievable on the Connectome scanner. However, due to recent advances in commercially available high‐gradient performance scanners, such as Siemens Cima.X (maximum gradient strength *G*_max_ = 200 mT/m) and GE MAGNUS (*G*_max_ = 300 mT/m) (Foo et al. 2020), it is possible to acquire diffusion data for the SANDI model on clinical scanners.

Furthermore, conventional clinical scanners already delivered the gradient performance needed for SANDI. For example, Schiavi et al. demonstrated a 10‐min, six *b*‐shell protocol (*b* = 500, 1000, 2000, 3000, 4000, 6000 s/mm^2^) on a 3 T Siemens Magnetom Prisma (specification *G*_max_ = 80 mT/m; implementation 67 mT/m) that yielded highly reproducible soma fraction maps in five healthy volunteers and five multiple sclerosis patients, confirming that the maximum *b*‐value up to 6000 s/mm^2^ is well within routine clinical reach (Schiavi et al. 2023). Similarly, Margoni et al. applied an almost identical sequence on a Philips Ingenia CX (*G*_max_ = 80 mT/m) in a cohort of 23 multiple sclerosis patients and 20 controls, confirming cross‐scanner robustness of soma fraction estimations (Margoni et al. 2023). At the same time, gradient technology is moving steadily upward. Together, these advances show that full SANDI acquisitions are now practical on routine clinical hardware and will become even more accessible as next‐generation high‐gradient systems enter mainstream use.

##### 3.1.8.5 Relationship Between DKI, NODDI, and SANDI Metrics

Diffusion metrics from different models can capture similar information of tissue microstructure, resulting in non‐trivial correlations between metrics from DKI, NODDI, and SANDI. For example, in the hippocampus of entire cohort (*n* = 72), DKI‐MD was positively correlated with NODDI‐*f*_iso_ (*r* = 0.81) and SANDI‐*f*_extra_ (*r* = 0.93), and DKI‐AK was positively correlated with NODDI‐ODI (*r* = 0.39). These correlations can be either analytically derived or numerically simulated, as shown in Supporting Information and Figures S10–S12.

### 3.2 Macroscopic and Microstructural Metrics Averaged Over the Lifespan: Analysis by Hippocampal Subregion

#### 3.2.1 Subiculum

Its low curvature reflects the nature of less surface wrapping compared to other subfields. The high *f*_icvf_ from the NODDI model, high FA from the DTI model, and high T1_w_/T2_w_ ratio (Figure S3) suggest a dense concentration of myelinated axons or dendrites and glia, similar to what has been observed in the averaged maps in young healthy subjects (Karat et al. 2023); this also aligns with the observed high *f*_neurite_ from the SANDI model. Further, the low *f*_soma_ and *R*_soma_ from the SANDI model in the body of the subiculum suggest fewer and smaller neuronal cell bodies; the high extracellular diffusivity (*D*_e_) and extracellular fraction (*f*_extra_) suggest a larger extracellular compartment. The specialized cellular architecture of the subiculum can be attributed to its role as a key output region from the hippocampus to other cortical areas (Böhm et al. 2018).

#### 3.2.2 CAs

In the CA regions, the thickness generally decreases, and curvature increases from CA1 to CA3. We believe that the SANDI metrics may effectively capture the organization of pyramidal cells, with CA1 showing higher *f*_soma_ and smaller *R*_soma_ compared to CA2–3, consistent with previous histological observations that CA1 shows smaller but densely packed cell bodies compared to CA2/3 (Dudek et al. 2016). Furthermore, in CA1/3, we observed higher *f*_neurite_ from the SANDI model and higher *f*_icvf_ from the NODDI model, compared with CA2. This coincides with previous histological results that CA1 has denser oblique dendritic organization (Dudek et al. 2016) and the presence of mossy fibers, mainly in CA3 (Insausti et al. 2023).

#### 3.2.3 Dentate Gyrus (DG)

The *f*_soma_ and *R*_soma_ values are the highest in the body, suggesting large and densely packed neurons in this region, likely due to the densely packed granule cells within DG (Bartsch and Arzy 2014; DeKraker et al. 2020), as also shown in Table 1. The high *D*_e_ and *f*_extra_ in DG's anterior and posterior poles indicate larger extracellular space.

### 3.3 Sex Differences in Aging Trajectory

Prior research has reported sex‐related differences in brain aging, such as findings of microstructural metrics in the brain white matter by Kochunov et al. (2012), Lawrence et al. (2021), Lebel et al. (2012), Mendez Colmenares et al. (2023), Ritchie et al. (2018), Szeszko et al. (2003), Toschi et al. (2020) and of macrostructural metrics in gray matter by Raz (1997); Wang et al. (2019); Xu et al. (2000). However, we found no significant sex effects on most of our hippocampal micro‐ and macro‐structural measures (“Age + Sex” model in Figure S6). In our dataset, the age distribution was balanced between 32 males (mean = 42.25 ± 17.99 years; median = 37.00 years) and 40 females (mean = 40.75 ± 17.98 years; median = 38.00 years), without significant differences in age distribution (two‐sample *t*‐test, *t*‐statistic: 0.3517, *p*‐value: 0.7261). Notably, a recent study of cortical regions using the same dataset (Lee, Lee, et al. 2024) likewise found no robust sex‐related effects. Sex‐related aging differences of microstructural metrics were neither reported nor observed in most previous literature on gray matter (Benedetti et al. 2006; Chan et al. 2024; Giorgio et al. 2010; Lee, Lee, et al. 2024; Ni et al. 2010; Pfefferbaum et al. 2010; Rathi et al. 2014; Singh et al. 2024), except in the deep gray matter, such as substantia nigra, red nucleus, putamen, and thalamus (Gong et al. 2014).

### 3.4 Limitations and Outlook

This study has several limitations.

We applied a self‐similarity‐based super‐resolution algorithm (Figures S8 and S9) and mid‐thickness surface sampling to reduce the partial‐volume effects from adjacent CSF or white matter, particularly near the hippocampal boundaries. When compared with results at the original resolution, the histograms of SANDI metrics with super‐resolution were narrower (Figure S9), potentially suggesting the reduction of partial‐volume contamination. To eliminate the partial volume effect, future studies could use advanced tissue‐type modeling or advanced diffusion MRI acquisitions using submillimeter spatial resolution (Dong et al. 2025) or hybrid approaches (Fan et al. 2017) to further mitigate these boundary artifacts.

Our analyses were cross‐sectional and did not include comprehensive cognitive testing or the years of education, limiting our ability to infer causal links between microstructural changes and cognitive decline. Longitudinal studies with cognitive assessment are needed to confirm the temporal sequence of these changes and to assess their impact on cognitive function more directly.

While the SANDI model provides detailed microstructural information, it relies on specific assumptions about tissue compartments that may not fully capture the complexity of hippocampal aging. For instance, the SANDI model's signal fractions are influenced by echo time (TE) due to varying T2 relaxation times across tissue compartments (Gong et al. 2023). This may introduce complexity in studying age‐related changes, as T2 variations—whether directly age‐related or not—can affect the estimated fractions. The SANDI model is currently unable to distinguish cell bodies of different cell types, such as neurons and glial cells. Given that glial cells are generally smaller in cell body size compared to neurons (von Bartheld 2018) as listed in Table 1, and the soma radius estimation in SANDI is skewed towards larger cell bodies (Afzali et al. 2021; Nilsson et al. 2017; Palombo et al. 2020), we expect that our observations mainly reflect changes in neuronal soma. Although we conducted a thorough literature review in Table 1, the availability of histological studies remained limited, and the techniques varied considerably across studies. The observed reduction in *R*_soma_ with aging could reflect neuronal cell body shrinkage (Mani et al. 1986) and/or a reduction in the neuron‐to‐glial ratio, as neurons generally decline in number with age (Šimić et al. 1997; West 1993), while glial cells tend to proliferate with age (De Lucia et al. 2016). Future studies should consider the complex cell composition in diffusion modeling (Garcia‐Hernandez et al. 2022).

A simple linear regression model can only capture monotonic trends, yet we employed it here to assess age‐related changes due to the constraints of our dataset (72 participants aged 19–85 years). The limited sample size and the lack of younger participants (i.e., the developmental range) made it challenging to draw robust conclusions from higher‐order polynomial regressions of age. In line with previous work reporting potential nonlinear age effects (Alsameen et al. 2023; Beck et al. 2021; Behler et al. 2021; Bouhrara et al. 2020, 2023; Kiely et al. 2022; Pietrasik et al. 2020; Qian et al. 2020; Singh et al. 2024; Slater et al. 2019; Storsve et al. 2016; Westlye et al. 2010), we tested whether an additional quadratic term (Age^2^) improved model fit for our data. Although the Age + Age^2^ model reached statistical significance in the T1_w_/T2_w_ metrics in some subfields (“M2: Age + Age^2^” in Figures S6 and S7), the overall model comparisons via likelihood ratio tests did not consistently favor the complicated model M2 over a simple linear (M1: Age‐only) specification in other metrics. Accordingly, given the current age span and number of subjects, we did not find compelling evidence to adopt a quadratic model—an outcome consistent with other studies using linear fits (Abe et al. 2008; Boban et al. 2022; Eikenes et al. 2023; Fjell et al. 2010; Fujita et al. 2023; Karat et al. 2024; Lee, Lee, et al. 2024; Mendez Colmenares et al. 2023; Merluzzi et al. 2016; Sullivan and Pfefferbaum 2006). Moreover, studies in the developing brain have reported different trajectories for hippocampal microstructural alterations (Karat et al. 2024), suggesting that a broader age span and larger sample size would be necessary to pinpoint the onset and progression of age‐related hippocampal changes using more complicated models.

Future studies in larger cohorts using the latest high‐gradient performance MRI scanner (*G*_max_ = 500 mT/m) (Huang et al. 2021; Ramos‐Llordén et al. 2025) will enable us to adopt higher‐order polynomial fitting or non‐parametric approaches (Chan et al. 2024; Rutherford et al. 2022) and provide a more comprehensive understanding of microstructural and macrostructural measures within the aging hippocampus. Furthermore, adapting an optimized dMRI technique for use on more widely available clinical‐grade MRI scanners with high‐performance gradients (Vachha and Huang 2021), such as the Siemens 3 T Cima.X scanner and the GE 3 T MAGNUS scanner (Foo et al. 2020) (both *G*_max_ ≥ 200 mT/m), will facilitate larger‐scale clinical research studies.

## 4 Conclusions

In this study, we combined high‐gradient diffusion MRI, the SANDI model, and HippUnfold hippocampal unfolding to detect subtle microstructural changes in aging. We observed region‐specific alterations in not just morphological but also microstructural metrics with age that likely reflect neuron shrinkage/loss and extracellular space expansion. These findings underline the importance of advanced diffusion acquisition, biophysical modeling, super‐resolution techniques, and structural unfolding algorithms in identifying distinct aging trajectories across hippocampal subfields.

## 5 Materials and Methods

### 5.1 Participants

A cohort of 72 participants (40 females: mean = 40.75 ± standard deviation 17.98 years; median = 38.00 years; 32 males: mean = 42.25 ± standard deviation 17.99 years; median = 37.00 years), aged between 19 and 85 years old (mean age 41.40 ± standard deviation 17.91 years, median = 37.00), was recruited and scanned for this study. The study protocol was approved by the institutional review board; all participants provided written informed consent prior to participation. The same cohort was used to study the SANDI metrics in the cortical regions (Lee, Lee, et al. 2024, Lee, Woo, et al. 2024). This study was a continuation of the previous one, focusing specifically on the hippocampal region.

### 5.2 Data Acquisition

Diffusion MRI data were acquired using a 3 T Connectome MRI scanner with maximal gradients of 300 mT/m and a 64‐channel head coil. The diffusion‐weighted imaging (DWI) protocol included monopolar pulsed‐gradient spin‐echo sequences with diffusion times (Δ) of 19 milliseconds and a fixed pulse duration (δ) of 8 ms. Imaging parameters included an echo time/repetition time (TE/TR) of 77/4000 ms and a 2 mm isotropic voxel size. The DWIs were acquired with eight *b*‐values ranging from 50 to 6000 s/mm^2^. The *b* = 0 images were included every 16 DWIs, using 32 gradient directions for *b*‐values below 2300 s/mm^2^ and 64 for higher *b*‐values. The total scan time was approximately 30 min. Additionally, multi‐echo magnetization‐prepared gradient echo (MEMPRAGE) T1‐weighted anatomical images were acquired at a 1 mm isotropic voxel size for hippocampal segmentation.

### 5.3 Diffusion Processing and Super‐Resolution Algorithms

The acquired 2‐mm DWI data underwent diffusion preprocessing steps (Tian et al. 2022). First, the diffusion‐weighted images were applied with the gradient non‐linearity correction using in‐house MATLAB codes (Fan et al. 2016) and the Connectome 1.0 gradient coil coefficients provided by Siemens. Then diffusion images went through susceptibility‐induced distortion correction (TOPUP function from FSL). Specifically, the TOPUP function was performed with the default settings except that the “‐warpres” parameters at each iteration were set to “20,16,14,12,10,6,4,2,2” to account for the 2‐mm isotropic resolution of our diffusion acquisition, and eddy‐current‐induced distortion correction (EDDY function from FSL) with dynamic susceptibility correction and slice‐to‐volume motion correction to optimally account for any potential motion (Andersson et al. 2016; Andersson and Sotiropoulos 2016).

As a final step in diffusion processing, a super‐resolution algorithm was employed to enhance the spatial resolution of the diffusion data. This algorithm leveraged high spatial frequency details from the deformed high‐resolution T1‐weighted images and translated them to the low‐resolution DWIs using self‐similarity, thereby maintaining high spatial fidelity and signal‐to‐noise ratio (SNR). (Coupé et al. 2013; Lee et al. 2019; Manjón et al. 2010).

In contrast to standard interpolation (e.g., cubic or trilinear), which may blur the image by simply blending neighboring voxel intensities based on distance (Tian et al. 2020), our super‐resolution approach leveraged local self‐similarities within a search volume to better preserve tissue boundaries and diffusion contrast. Specifically, we defined a local search volume (sliding window), where interpolation weights of self‐similarity were computed based on signal intensity differences between the target voxel and neighboring voxels in both DWI and co‐registered T1‐weighted images. Distance between voxels was not considered in the self‐similarity weight. The super‐resolution image is reconstructed by using interpolation weights from signal intensity differences in the local search volume, ensuring that the algorithm can tolerate a small misregistration of up to 1–2 mm (Manjón et al. 2010). Further, we enforce data fidelity at each iteration by requiring that the down‐sampled version of the super‐resolved image remained consistent with the original low‐resolution DWI. This constraint ensured data consistency and convergence of the algorithm over a few iterations, preserving the diffusion contrast in the original low‐resolution DWI. The super‐resolution technique mitigated partial‐volume effects, yielding sharper tissue boundaries and a more accurate representation of hippocampal microstructure.

The following paragraphs provide the technical details of this implementation, including the iterative weighting scheme, data consistency enforcement, and parallelization strategies.

The super‐resolution algorithm was implemented in‐house as the “mrsupres” function (https://github.com/yixinma9/super_resolution/) compatible with the MRtrix3 toolbox (Tournier et al. 2019). Initially, low‐resolution 2‐mm DWIs were linearly interpolated to 1‐mm resolution using the “mrgrid” function with nearest neighbor, providing the initial input for the “mrsupres” function. High‐resolution T1‐weighted image was registered to the upsampled diffusion‐weighted images using “bbregister” (Greve and Fischl 2009) to ensure accurate spatial alignment. Aligned high‐resolution T1w image was used as a reference image.

For each voxel in the DWI, weights were computed within a 5 × 5 × 5 kernel. At each iteration *t*, the weights were calculated based on the similarity between 3 × 3 × 3 patches of the DWI and the corresponding T1w image voxels. For example, for a voxel at position (i, j, k), with (x, y, z) being one of the neighboring voxels in the 5 × 5 × 5 kernel, the specific weight was calculated using the following function:

$$\begin{array}{c} -\ln\left(w_{\left[x,y,z\right]}^{t}\right)=\frac{\sum_{\text{patch}}{\left({\mathrm{DWI}}_{\left[x_{p},y_{p},z_{p}\right]}^{t}-{\mathrm{DWI}}_{\left[i_{p},j_{p},k_{p}\right]}^{t}\right)}^{2}\cdot h^{2}}{3^{3}\times k_{\text{DWI}}}\\ \;+\frac{{\left(T{1_{w}}_{\left[x,y,z\right]}-T{1_{w}}_{\left[i,j,k\right]}\right)}^{2}\cdot h^{2}}{k_{{\text{T1}}_{\text{w}}}} \end{array}$$

where *h* was a hyper‐parameter that increased with each iteration; a series *h* of 1, 2, 4, 6, 8, 16 was adopted for six iterations here. [Correction added on 03 December 2025 after first online publication: The numerator in the equation and “h‐series numbers” in the subsequent paragraph have been corrected.] The normalization factors *k*_DWI_ and *k*_T1w_ were mean square signals of DWI and T1w image within the brain mask, respectively. The (*i*_*p*_, *j*_*p*_, *k*_*p*_) and (*x*_*p*_, *y*_*p*_, *z*_*p*_) were voxels within the 3 × 3 × 3 patches centered at (*i*, *j*, *k*) and (*x*, *y*, *z*) with the same shifts, respectively, i.e., *i*_*p*_ ‐ *i* = *x*_*p*_ ‐ *x*, *j*_*p*_ ‐ *j* = *y*_*p*_ ‐ *y*, and *k*_*p*_ ‐ *k* = *z*_*p*_ ‐ *z*. The high‐resolution DWI at next iteration was computed as following:

$${\text{DWI}}_{\left[i,j,k\right]}^{t+1}=\frac{\sum_{\text{kernel}}w_{\left[x,y,z\right]}^{t}\times {\text{DWI}}_{\left[x,y,z\right]}^{t}}{\sum_{\text{kernel}}w_{\left[x,y,z\right]}}$$

Data consistency in this inverse problem was enforced by adding back the residual difference between the down‐sampled high‐resolution estimate and the original low‐resolution image. This ensured that after each iteration of the super‐resolution process, the high‐resolution image, when downsampled, remained consistent with the original low‐resolution image. By iteratively refining the high‐resolution estimate and ensuring consistency with the original data, the algorithm effectively enhanced the resolution of DWIs while preserving the fidelity of the original measurements.

Moreover, the “mrsupres” function was designed to leverage parallel computing, enhancing performance significantly. Key computational steps described above, including weight calculation, intensity update, and data consistency adjustment, were parallelized using multi‐threading. This approach distributed the computational load across multiple CPU cores, allowing for efficient processing of large‐scale datasets.

The hippocampus is small and highly convoluted, making it susceptible to partial volume effects when using 2 mm isotropic diffusion‐weighted images. By applying a self‐similarity‐based super‐resolution approach with a local search volume, we reduced the partial volume contamination and enhanced spatial fidelity. This was critical for capturing finer subfield‐level variations of morphological and microstructural features in aging populations.

### 5.4 Hippocampal Segmentation Using HippUnfold and the Structural Metrics

**FIGURE 5:**
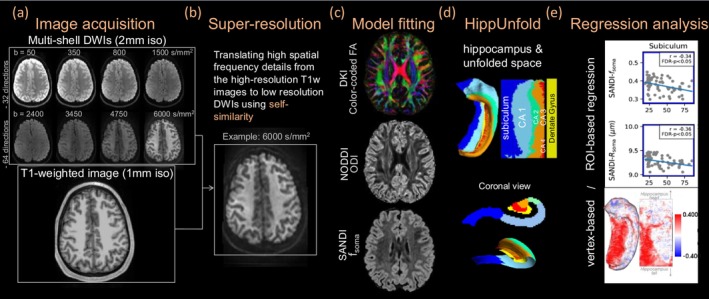
Image acquisition and analysis workflow. (a) Multi‐shell diffusion‐weighted images (DWIs) and high‐resolution T1‐weighted images were acquired for all subjects. (b) Super‐resolution processing of DWIs using high‐resolution T1‐weighted images. (c) Diffusion model fitting, including DKI, NODDI, and SANDI metrics. (d) Hippocampal segmentation using HippUnfold. (e) Regression analysis assessing age‐related changes in structural and diffusion metrics at ROI and vertex levels.

Hippocampal segmentation was performed using the HippUnfold toolbox (DeKraker et al. 2020, 2022). The hippocampus was segmented into six distinct regions of interest (ROIs), including the subiculum, CA1–CA4, and dentate gyrus (DG). Figure 5d illustrated the folding of the hippocampal gray matter and the corresponding ROIs. Structural metrics at each vertex on the mid‐thickness hippocampal surface were generated using this toolbox, where thickness was defined as the distance between the inner and outer surfaces of the hippocampal gray matter. Since the dentate gyrus marks the end of the hippocampal folding, the dentate gyrus did not have a two‐layer structure (inner and outer surfaces), and thus thickness was not defined for the dentate gyrus.

Curvature was quantified by calculating the mean curvature (H), which was the average of the principal curvatures at each vertex: $H=\frac{k_{1}+k_{2}\;}{2}$, where *k*_1_ and *k*_2_ were the principal curvatures at each vertex, computed automatically by HippUnfold. Gyrification was defined as the ratio of the surface area in the native space to the surface area in the unfolded space. These macroscopic metrics were subsequently used in regression analyses with age.

### 5.5 Diffusion Model Fitting

The SANDI metrics were calculated by fitting the spherical mean signals of multi‐shell DWIs using the SANDI MATLAB toolbox (available at https://github.com/palombom/SANDI‐Matlab‐Toolbox). This toolbox used a random forest regression model with MATLAB's TreeBagger function (200 trees). The following parameter choices followed the original SANDI publication (Palombo et al. 2020) and our prior experience in applying the SANDI model in the cortical gray matter region (H. Lee, Lee, et al. 2024, Lee, Woo, et al. 2024). During training, the intra‐soma diffusivity (*D*_is_) was fixed at 3 μm^2^/ms, while five other parameters were uniformly sampled within predefined ranges: extracellular diffusivity (*D*_e_) between 0.25 and 3 μm^2^/ms, intra‐neurite diffusivity (*D*_in_) between 0.25 and 3 μm^2^/ms, soma radius (*R*_soma_) from 1 to 12 μm, intra‐neurite signal fraction (*f*_neurite_) from 0 to 1, and intra‐soma signal fraction (*f*_soma_) from 0 to 1. The training dataset consisted of normalized simulated signals generated from 100,000 different parameter combinations. To estimate the signal‐to‐noise ratio (SNR) for the *b* = 0 images, we first applied Marchenko‐Pastur principal component analysis (MP‐PCA) denoising to the raw MRI images using “dwidenoise” from Mrtrix3 (Tournier et al. 2019; Veraart et al. 2013, 2016). The noise map was then corrected for gradient non‐linearity (Fan et al. 2016). The noise map was squared before up‐sampling to account for the increase in variance due to the addition of independent and identically distributed (i.i.d.) noise at each voxel. The extracellular signal fraction (*f*_extra_) was calculated under the constraint that *f*_soma_ + *f*_neurite_ + *f*_extra_ = 1, which differed from the original study (Palombo et al. 2020), where the constraint was *f*_soma_ + *f*_neurite_ = 1.

Diffusion Kurtosis Imaging (DKI) model fitting was performed using the MRtrix3 toolbox (Tournier et al. 2019). Multi‐shell diffusion‐weighted images (DWIs) of *b*‐values of 0, 800, 1500, 2400, and 3450 s/mm^2^ were fitted using the “dwi2tensor” command with the “‐constrain” option to enforce the non‐negative apparent diffusivity and kurtosis and monotonic signal decay (Morez et al. 2023; Tournier et al. 2019; Veraart et al. 2013). Finally, Neurite Orientation Dispersion and Density Imaging (NODDI) (Zhang et al. 2012) metrics were derived using the NODDI Matlab toolbox, available at http://mig.cs.ucl.ac.uk/index.php?n=Tutorial.NODDImatlab.

### 5.6 ROI‐Based Regression Analysis

Diffusion metrics were sampled at each vertex on the mid‐thickness of the hippocampal surface to avoid partial volume effect. Structural metrics were precomputed at each vertex using the HippUnfold toolbox. For each subject, the average of these metrics across left and right hippocampal mid‐thickness surfaces was calculated. A linear regression model was used to investigate the relationship between each metric and age, with biological sex included as an additional covariate. The regression model was expressed as:

Metric=β0+β1×Age+β2×Biologicalsex+ϵ

where β0 represented the intercept, β1 and β2 were the regression coefficients for age and gender, respectively, and ϵ was the error term. The null hypothesis (H0: β1 = 0) posited no association between age and the metric, while the alternative hypothesis (H1: β1 ≠ 0) suggested a significant relationship. The *t*‐statistic was used to test these hypotheses. *p*‐values from the *t*‐test on β1 were corrected for multiple comparisons using the False Discovery Rate (FDR) following the Benjamini‐Hochberg procedure, which adjusted *p*‐values to reduce the likelihood of false positives, ensuring that the reported significant associations are statistically robust.

After running the multivariate regression, the second covariate (biological sex) was found to be non‐significant for all metrics. Therefore, the focus shifted to the primary variable of interest: age. To simplify the analysis, a simple linear regression was conducted with age as the sole predictor, which corresponds directly to reporting the Pearson correlation coefficient (*r*). This is because, in the case of one‐variable linear regression, the correlation coefficient *r* captures the strength and direction of the linear relationship between age and each metric. It's also worth noting that under one‐variable linear regression *r* monotonically reflect *t*‐score: $t=\frac{r\;\sqrt{n-2}}{\sqrt{1-r^{2}}}$ with the sample size *n*.

### 5.7 Vertex‐Based Analysis

We began by calculating and displaying the mean values of structural and SANDI metrics across all subjects, spanning the full age range, on the unfolded hippocampal surface. These mean metrics, averaged across subjects of all ages, were shown in Figure 3 and Figure S2, providing a baseline overview of the distribution of these metrics across the hippocampal surface.

Next, we performed vertex‐based regression analyses to visualize how diffusion metrics change with age. This involved applying the linear regression model at each vertex for all subjects, allowing us to assess localized, age‐related changes across the hippocampal mid‐thickness surface. The HippUnfold toolbox facilitated the projection of all subjects onto a common template, enabling consistent comparison across individuals. T‐score of β1 from regression: Metric = β0 + β1 × Age + β2 × biological sex + ϵ is displayed in Figures S4 and S5; correlation between metrics and age displayed in Figure 3 and Figure S3, both showing the age‐dependent alterations in structural and diffusion metrics, highlighted vertices with substantial positive or negative relationships with age.

Together, the analyses of mean values and age‐dependent alterations offer a comprehensive understanding of both the overall distribution of structural and diffusion metrics and their changes across the hippocampal surface with aging.

### 5.8 Correlation Matrix Analysis

We computed a Pearson correlation matrix to examine the relationships between various structural and diffusion metrics across the hippocampal surface. For each pair of metrics, we compared their values across all vertices to assess the degree of their linear covariation using the Numpy “corr” function. The resulting correlation matrix highlighted the spatial associations between metrics across the hippocampal surface, with significance indicated after FDR multiple correction. These matrices, presented in Figures 3 and 4 and Figures S2 and S3, provided insights into the relationships between metrics, both for the mean values and the age‐related changes.

## Author Contributions

**Yixin Ma:** conceptualization, data curation, formal analysis, investigation, methodology, validation, visualization, writing – original draft, and writing – review and editing. **Hansol Lee:** data collection, data curation, formal analysis, validation, and writing – review and editing. **Kwok‐Shing Chan:** formal analysis, validation, and writing – review and editing. **Laleh Eskandarian:** data curation, formal analysis, validation, and writing – review and editing. **Kyla Gaudet:** data collection, data curation, and formal analysis. **Qiyuan Tian:** formal analysis, funding acquisition, and writing – review and editing. **Aneri Bhatt:** data collection, data curation, and formal analysis. **Julianna Gerold:** data collection, data curation, and formal analysis. **Andrew W. Russo:** data collection, data curation, and formal analysis. **David H. Salat:** conceptualization, funding acquisition, and writing – review and editing. **Eric C. Klawiter:** conceptualization, funding acquisition, and writing – review and editing. **Susie Y. Huang:** conceptualization, data collection, investigation, funding acquisition, project administration, resources, supervision, and writing – review and editing. **Hong‐Hsi Lee:** conceptualization, data collection, investigation, funding acquisition, project administration, resources, supervision, and writing – review and editing.

## Conflicts of Interest

The authors declare no conflicts of interest.

## Supporting information


**Figure S1:** Age‐related changes in NODDI, DKI, and T1_w_/T2_w_ metrics. Scatter plots showing age‐related changes in NODDI, DKI, and T1_w_/T2_w_ metrics across hippocampal subfields, with significant correlations marked after FDR correction.
**Figure S2:** Mean values of NODDI, DKI, and T1_w_/T2_w_ Metrics on the unfolded hippocampal surface. Mean values of NODDI, DKI, and T1_w_/T2_w_ metrics displayed on the hippocampal surface, highlighting the spatial distribution across the subfields.
**Figure S3:** Age‐related Changes in NODDI, DKI, and T1_w_/T2_w_ Metrics. Age‐related correlation coefficients for NODDI, DKI, and T1_w_/T2_w_ metrics on the unfolded hippocampal surface, with significant correlations highlighted and spatial relationships shown in the correlation matrix.
**Figure S4:** The figure displays *t*‐statistics mapped onto hippocampal subfields, showing the association between age and structural and SANDI metrics. The regression model is Metric = β0 + β1 × Age + β2 × Gender + ϵ, where β1 reflects the effect of age. Red areas indicate positive associations (*t* > 0) and blue areas negative (*t* < 0). The *t*‐statistics test the null hypothesis (H0: β1 = 0).
**Figure S5:** The figure displays *t*‐statistics mapped onto hippocampal subfields, showing the association between age and DKI, NODDI, and T1_w_/T2_w_ metrics. The regression model is Metric = β0 + β1 × Age + β2 × Gender + ϵ, where β1 reflects the effect of age. Red areas indicate positive associations (*t* > 0) and blue areas negative (*t* < 0). The *t*‐statistics test the null hypothesis (H0: β1 = 0).
**Figure S6:** Heatmaps of regression *z*‐scores for four models of hippocampal metric values as a function of age, age^2^ and/or sex. Columns represent: M1: Age; M2: Age + Age^2^; M3: Age + Sex; M4: Age + Age^2^ + Sex; and M5: Age + ICV (Intra‐Cranial Volume). Each cell shows the *z*‐score of the corresponding parameter (rows are parameters, columns are hippocampal subfields), with positive values in red and negative values in blue. Cells marked by a star (*) indicate statistical significance with FDR‐*p* < 0.05.
**Figure S7:** Likelihood ratio tests (LRT) *p*‐values for comparing M1 (Age‐only model) against each more complex model: M2 (Age + Age^2^), M3 (Age + Sex), and M4 (Age + Age^2^ + Sex). Each column displays the *p*‐values across hippocampal metrics (rows) and subfields (columns), color‐coded from 0 (cyan) to 1 (magenta). Cells marked with a star (*) are those where the LRT with *p* < 0.05, indicating that the more complicated model provides a significantly better fit than M1 for that metric and subfield.
**Figure S8:** Comparison of SANDI metrics sampled and displayed on the HippUnfold space in a single representative subject, without (left panel, 2 mm isotropic) and with (right panel, 1 mm isotropic) super‐resolution processing. Super‐resolution processing enabled revealing of more detailed microstructural variations within the hippocampal subfields compared to the original resolution.
**Figure S9:** Histograms of super‐resolution vs. original (low) resolution SANDI metrics in the hippocampus of one subject. These distributions illustrate how the super‐resolution reconstruction reduces the partial volume effect, leading to narrower histograms. Separate tables showing four key statistical measures for each diffusion metric (range 95%–5%, interquartile range 75%–25%, mean, and standard deviation).
**Figure S10:** Scatter plot shows a positive correlation between orientation dispersion index (ODI, from NODDI) and axial kurtosis (AK, from DKI). Spearman correlation coefficients (R) of 0.7187 quantifies their positive correlation.
**Figure S11:** Voxelwise Pearson correlation matrix between diffusion kurtosis imaging (DKI) metrics and biophysical model metrics derived from SANDI and NODDI in the bilateral hippocampus (averaged over 72 subjects). Diffusion metrics including DKI (AD, RD, MD, AK, RK, MK, FA), SANDI (*f*_soma_, *R*_soma_, *f*_extra_, *D*_e_, *f*_neurite_, *D*_in_) and NODDI (*f*_icvf_, *f*_iso_, ODI) are incorporated in the correlation analysis. Consistent with theoretical predictions, positive correlations emerge between MD and both NODDI‐*f*_iso_ and SANDI‐*f*_extra_, and AK shows a strong positive association with ODI. These empirical relationships corroborate the analytical and simulationbased analysis presented in Figure S10.
**Figure S12:** Voxel‐wise correlations between diffusion metrics: DKI‐MD vs. NODDI‐*f*_iso_, DKI‐MD vs. SANDI‐*f*_extra_ and DKI‐AK vs. NODDI‐ODI. Color indicates relative voxel density, with Pearson's R shown in each panel. Analyses were performed across all voxels from both hippocampi in all subjects.

## Data Availability

The data that support the findings of this study are available on request from the corresponding author. The data are not publicly available due to privacy or ethical restrictions.
