# Correction to “Age‐Related Alterations in Hippocampal Microstructure Quantified Using High‐Gradient Diffusion MRI (dMRI) in an Unfolded Hippocampal Space”

**DOI:** 10.1111/acel.70654

**Published:** 2026-08-02

Ma, Y. , H. Lee , K.‐S. Chan , et al. 2026. “Age‐Related Alterations in Hippocampal Microstructure Quantified Using High‐Gradient Diffusion MRI (dMRI) in an Unfolded Hippocampal Space.” Aging Cell 25, no. 1: e70274. 10.1111/acel.70274.41194528 PMC12740091

The Conflicts of Interest section should be updated as follows:

## Conflicts of Interest

Dr. Salat has a financial interest in Niji Corp.

We apologize for this error.

