# Supplementary material for: Age‐Related Alterations in Hippocampal Microstructure Quantified Using High‐Gradient Diffusion MRI (dMRI) in an Unfolded Hippocampal Space
Source: Aging Cell. 2025 Nov 5;25(1):e70274. doi: 10.1111/acel.70274 (PMC12740091; doi:10.1111/acel.70274)
Supplement: Supplementary file 1 — Figure S1: Age‐related changes in NODDI, DKI, and T1w/T2w metrics. Scatter plots showing age‐related changes in NODDI, DKI, and T1w/T2w metrics across hippocampal subfields, with significant correlations marked after FDR correction. Figure S2: Mean values of NODDI, DKI, and T1w/T2w Metrics on the unfolded hippocampal surface. Mean values of NODDI, DKI, and T1w/T2w metrics displayed on the hippocampal surface, highlighting the spatial distribution across the subfields. Figure S3: Age‐related Changes in NODDI, DKI, and T1w/T2w Metrics. Age‐related correlation coefficients for NODDI, DKI, and T1w/T2w metrics on the unfolded hippocampal surface, with significant correlations highlighted and spatial relationships shown in the correlation matrix. Figure S4: The figure displays t‐statistics mapped onto hippocampal subfields, showing the association between age and structural and SANDI metrics. The regression model is Metric = β0 + β1 × Age + β2 × Gender + ϵ, where β1 reflects the effect of age. Red areas indicate positive associations (t > 0) and blue areas negative (t < 0). The t‐statistics test the null hypothesis (H0: β1 = 0). Figure S5: The figure displays t‐statistics mapped onto hippocampal subfields, showing the association between age and DKI, NODDI, and T1w/T2w metrics. The regression model is Metric = β0 + β1 × Age + β2 × Gender + ϵ, where β1 reflects the effect of age. Red areas indicate positive associations (t > 0) and blue areas negative (t < 0). The t‐statistics test the null hypothesis (H0: β1 = 0). Figure S6: Heatmaps of regression z‐scores for four models of hippocampal metric values as a function of age, age2 and/or sex. Columns represent: M1: Age; M2: Age + Age2; M3: Age + Sex; M4: Age + Age2 + Sex; and M5: Age + ICV (Intra‐Cranial Volume). Each cell shows the z‐score of the corresponding parameter (rows are parameters, columns are hippocampal subfields), with positive values in red and negative values in blue. Cells marked by a star (*) indicate statis [file ACEL-25-e70274-s001.docx]

**Age-related alterations in hippocampal microstructure quantified using high-gradient diffusion MRI (dMRI) in an unfolded hippocampal space**

Yixin Ma^1,†^, Hansol Lee^1,2†^, Kwok-Shing Chan^1^, Laleh Eskandarian^1^, Kyla Gaudet^1^, Qiyuan Tian^1^, Aneri Bhatt^1^, Julianna Gerold^1^, Andrew W. Russo^3^, David H. Salat^1^, Eric C. Klawiter^3^, Susie Y. Huang^1,4^, and Hong-Hsi Lee^1,*^

^1^Athinoula A. Martinos Center for Biomedical Imaging, Department of Radiology, Massachusetts General Hospital, Charlestown, Massachusetts, USA

^2^Department of Biomedical Engineering, Ulsan National Institute of Science and Technology, Ulsan, South Korea

^3^Department of Neurology, Massachusetts General Hospital, Harvard Medical School, Boston, Massachusetts, USA

^4^Harvard-MIT Division of Health Sciences and Technology, Massachusetts Institute of Technology, Cambridge, Massachusetts, USA

**Running title:** Age-related hippocampal microstructure changes

**Keywords:** aging, hippocampal microstructure, diffusion MRI (dMRI), SANDI model, super-resolution, HippUnfold

† Yixin Ma and Hansol Lee contributed equally to this work as first authors.

* Corresponding author:

Hong-Hsi Lee

Address: Department of Radiology, Athinoula A. Martinos Center for Biomedical Imaging, Massachusetts General Hospital, 149 13th Street, Charlestown, Massachusetts 02129, USA

E-mail: [hlee84@mgh.harvard.edu](mailto:hlee84@mgh.harvard.edu)

**Supplementary Materials:**

**Cellular Characteristics and Age-Related Changes in Hippocampal Subregions**

This section outlines the methods used to summarize cellular characteristics and age-related changes in hippocampal subregions in Table 1. Data came from several studies, with estimates made as needed.

**Soma Radius Estimation**: When only cell body area (A) was available, we estimated the soma radius (r) using $r=\sqrt{\frac{A}{\pi}}$. For example, CA1 pyramidal neurons had an area of 350 μm² (Benavides-Piccione et al., 2019), and mossy cells ranged from 502.2 to 599.5 μm² (Grovola et al., 2020). Reported diameters, such as 15.6 µm for subiculum (Rosenblum et al., 2025), 25 μm for CA2-3, 15 μm for CA1 pyramidal cells, 8-15 µm for granule cells (Spencer & Bland, 2007), 7.6-16.6 µm for astrocytes (Bedner et al., 2019), and 2.5-16.6 µm for all kinds of microglia (López‐Meraz & Álvarez‐Croda, 2023), were converted to radii by dividing by two.

**Neuron Counts and Density Calculations:** Neuron counts were primarily obtained from Harding et al. (1998), Table 3, and West et al. (1994), Table: "Analysis of Age-Related and AD-Related Neuronal Loss in the Human Hippocampus." Harding et al. also reported the volume of each subfield in Table 1, allowing densities to be calculated by dividing cell counts by the corresponding subregion volumes.

**Supplementary Figure S1: Age-related changes in NODDI, DKI, and T1_w_/T2_w_ metrics.** Scatter plots showing age-related changes in NODDI, DKI, and T1_w_/T2_w_ metrics across hippocampal subfields, with significant correlations marked after FDR correction.

**Supplementary Figure S2: Mean values of NODDI, DKI, and T1_w_/T2_w_ Metrics on the unfolded hippocampal surface.** Mean values of NODDI, DKI, and T1_w_/T2_w_ metrics displayed on the hippocampal surface, highlighting the spatial distribution across the subfields.

**Supplementary Figure S3: Age-related Changes in NODDI, DKI, and T1_w_/T2_w_ Metrics.** Age-related correlation coefficients for NODDI, DKI, and T1_w_/T2_w_ metrics on the unfolded hippocampal surface, with significant correlations highlighted and spatial relationships shown in the correlation matrix.

**Supplementary Figure S4:**The figure displays t-statistics mapped onto hippocampal subfields, showing the association between age and structural and SANDI metrics. The regression model is Metric = β0 + β1 × Age + β2 × Gender + ϵ, where β1 reflects the effect of age. Red areas indicate positive associations (t > 0) and blue areas negative (t < 0). The t-statistics test the null hypothesis (H0: β1 = 0)

**Supplementary Figure S5:**The figure displays t-statistics mapped onto hippocampal subfields, showing the association between age and DKI, NODDI, and T1_w_/T2_w_ metrics. The regression model is Metric = β0 + β1 × Age + β2 × Gender + ϵ, where β1 reflects the effect of age. Red areas indicate positive associations (t > 0) and blue areas negative (t < 0). The t-statistics test the null hypothesis (H0: β1 = 0).

**Supplementary Figure S6.** Heatmaps of regression *z*‐scores for four models of hippocampal metric values as a function of age, age^2^ and/or sex. Columns represent: M1: Age; M2: Age + Age^2^; M3: Age + Sex; M4: Age + Age^2^ + Sex; and M5: Age + ICV (Intra-Cranial Volume). Each cell shows the *z*‐score of the corresponding parameter (rows are parameters, columns are hippocampal subfields), with positive values in red and negative values in blue. Cells marked by a star (*) indicate statistical significance with FDR-*p*<0.05.


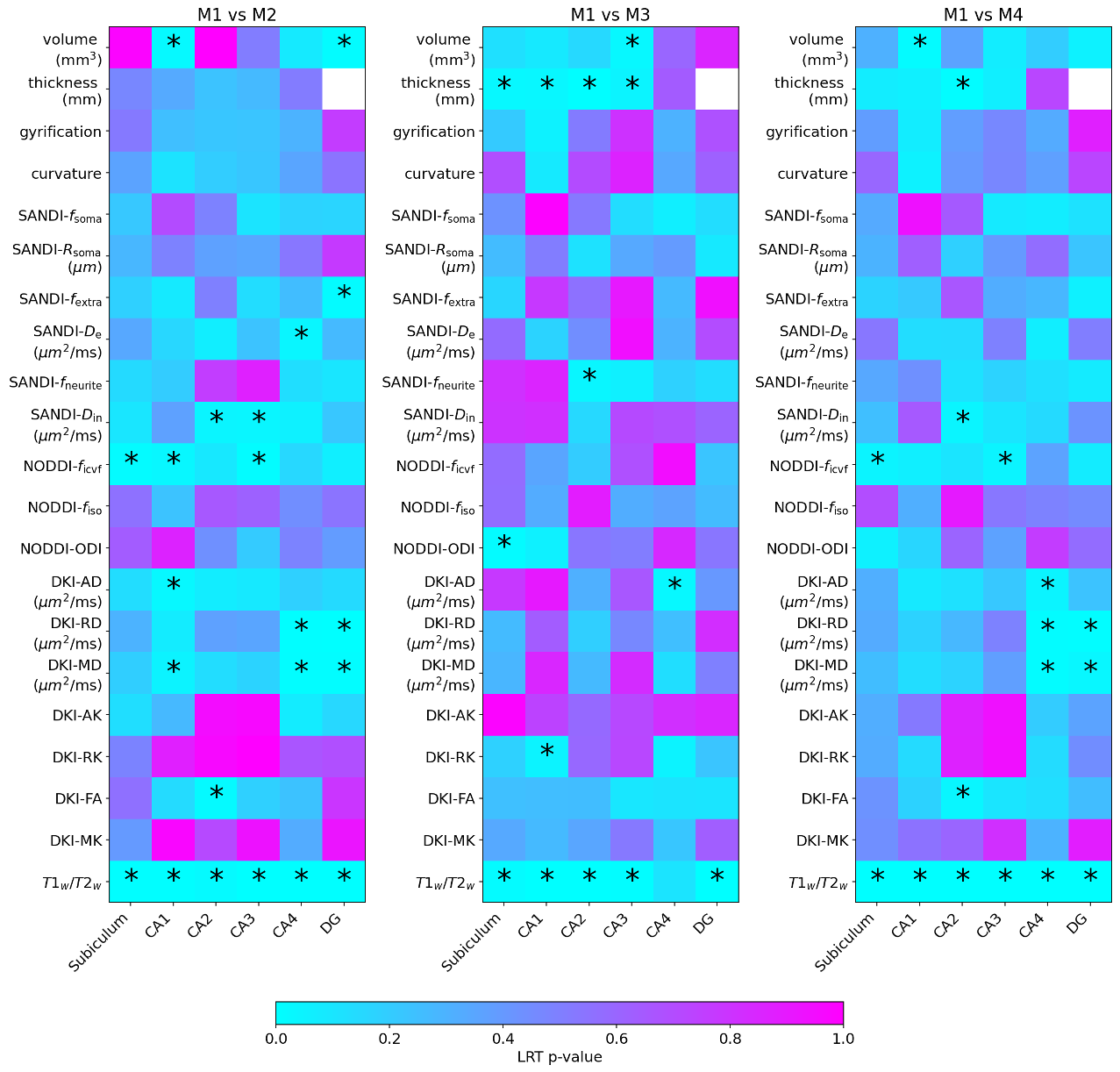


**Supplementary Figure S7.** Likelihood ratio tests (LRT) *p*‐values for comparing M1 (Age‐only model) against each more complex model: M2 (Age + Age^2^), M3 (Age + Sex), and M4 (Age + Age^2^ + Sex). Each column displays the *p*‐values across hippocampal metrics (rows) and subfields (columns), color‐coded from 0 (cyan) to 1 (magenta). Cells marked with a star (*) are those where the LRT with *p* < 0.05, indicating that the more complicated model provides a significantly better fit than M1 for that metric and subfield.


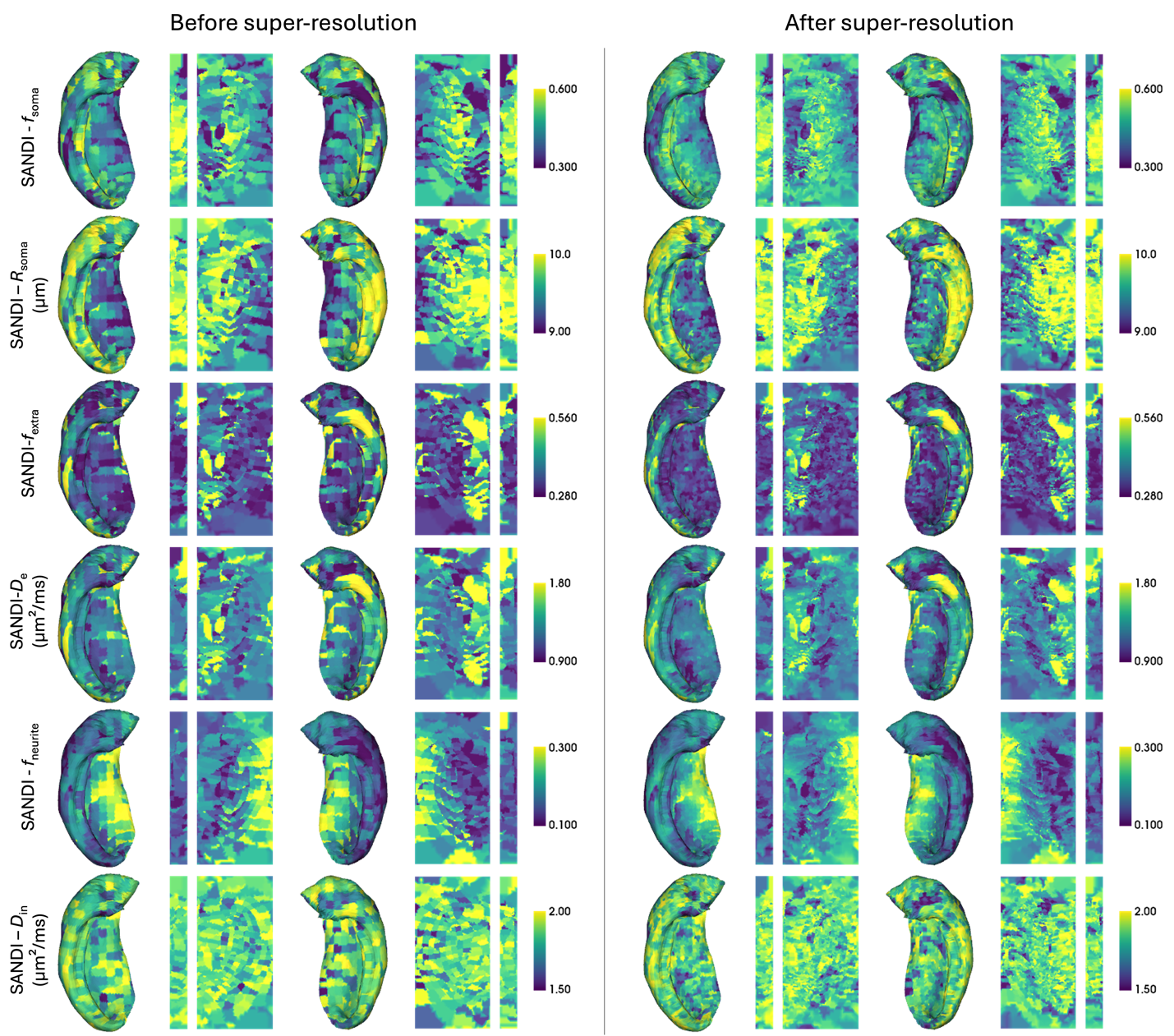


**Supplementary Figure S8:** Comparison of SANDI metrics sampled and displayed on the HippUnfold space in a single representative subject, without (left panel, 2 mm isotropic) and with (right panel, 1 mm isotropic) super-resolution processing. Super-resolution processing enabled revealing of more detailed microstructural variations within the hippocampal subfields compared to the original resolution.

1. Histogram of diffusion metrics on hippocampal vertices


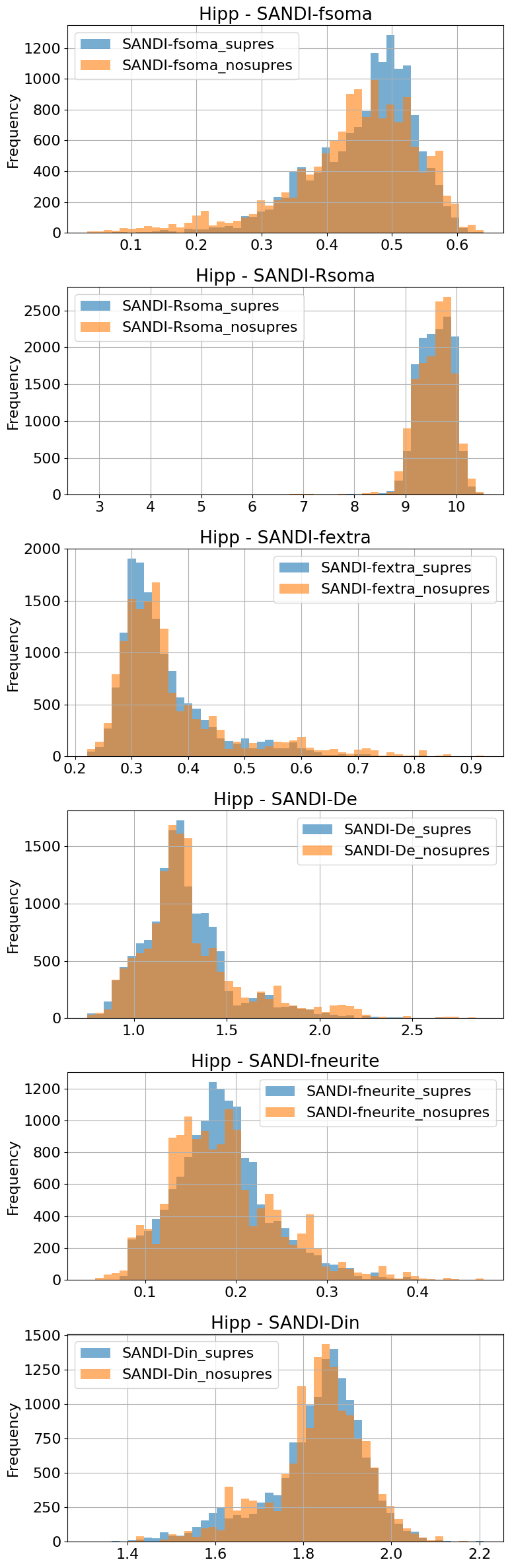


(b) statistics on super-resolution (1 mm isotropic)


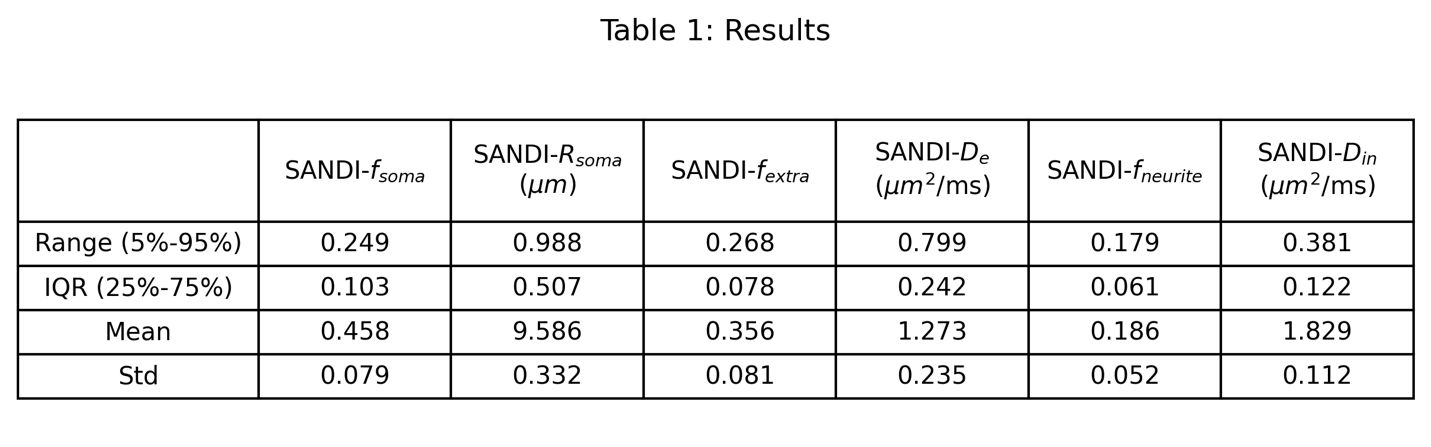


(c) Statistics on original (low) resolution (2 mm isotropic)


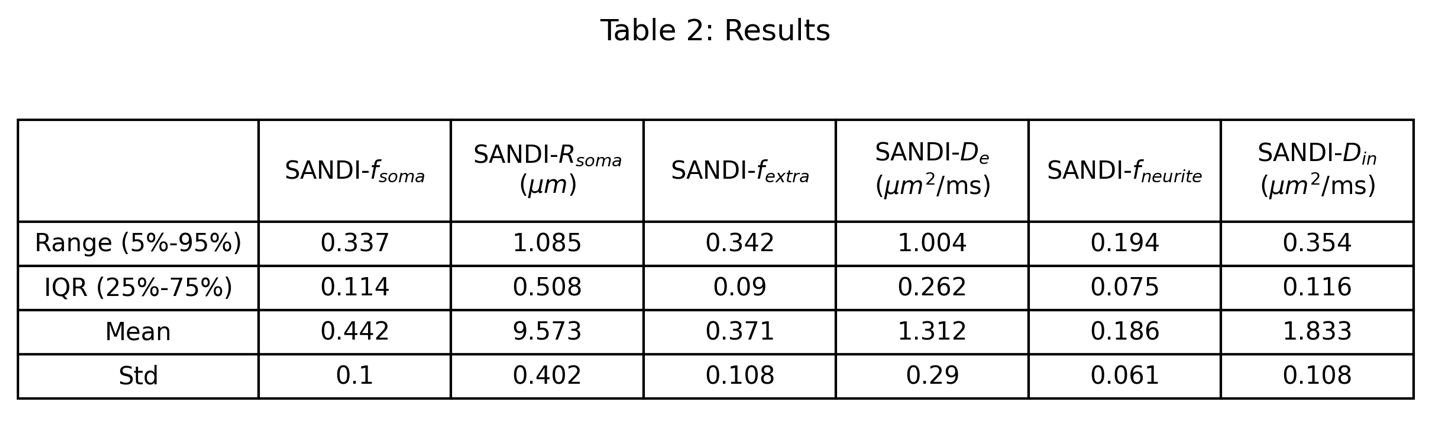


**Supplementary Figure S9:** Histograms of super-resolution vs. original (low) resolution SANDI metrics in the hippocampus of one subject. These distributions illustrate how the super-resolution reconstruction reduces the partial volume effect, leading to narrower histograms. Separate tables showing four key statistical measures for each diffusion metric (range 95%–5%, interquartile range 75%–25%, mean, and standard deviation).

**Correlations between metrics of DKI and biophysical models (NODDI, SANDI):**

Diffusion metrics from different models can capture similar information of tissue microstructure, resulting in non-trivial correlations between DKI-MD, NODDI-*f*_iso_, and SANDI-*f*_extra_ (Subiculum ROI), as well as NODDI-ODI and DKI-AK (CA1 ROI). These correlations can be either analytically derived or numerically simulated as follows.

In NODDI model, intra-axonal axial diffusivity $D_{a}$ and extracellular axial diffusivity $D_{e}^{\parallel}$ were both fixed at 1.7 µm^2^/ms, and free water diffusivity $D_{\mathrm{iso}}$ was fixed at 3 µm^2^/ms. The extracellular radial diffusivity was defined by using a tortuosity relation $D_{e}^{\perp}=\left( 1-f_{\mathrm{icvf}} \right)\cdot D_{e}^{\parallel}$ with an intra-axonal volume fraction $f_{\mathrm{icvf}}$. For a multi-compartmental model, the overall MD is the volume-weighted sum of MD in each compartment (Novikov et al., 2018). Therefore, the NODDI model has an equivalent MD, given by

$$MD=\left( 1-f_{\mathrm{iso}} \right)\cdot\left[ f_{\mathrm{icvf}}\cdot\frac{1}{3}D_{a}+\left( 1-f_{\mathrm{icvf}} \right)\cdot\left( \frac{1}{3}D_{e}^{\parallel}+\frac{2}{3}D_{e}^{\perp} \right) \right]+f_{\mathrm{iso}}\cdot D_{\mathrm{iso}}$$

$$D_{a}=D_{e}^{\parallel}=1.7 \mu m^{2}/\mathrm{ms}, D_{e}^{\perp}=\left( 1-f_{\mathrm{icvf}} \right)\cdot D_{e}^{\parallel}$$

Based on the above relations, the DKI-MD and NODDI-*f*_iso_ have a positive correlation due to

$$\frac{\partial MD}{\partial f_{\mathrm{iso}}}=-D_{a}\cdot\frac{1+2\left( 1-f_{\mathrm{icvf}} \right)^{2}}{3}+D_{\mathrm{iso}}\geq-D_{a}+D_{\mathrm{iso}}>0,$$

aligning with the observation.

Furthermore, SANDI model is composed of intra-neurite, intra-soma, and extra-cellular space. Intra-neurite space is modeled as sticks with volume fraction $f_{\mathrm{neurite}}$ and diffusivity along neurites $D_{\mathrm{neurite}}$. Intra-soma space is modeled as an isotropic restricted diffusion with apparent diffusivity $\mathrm{AD}C_{\mathrm{soma}}$. Extracellular space is modeled as an isotropic Gaussian diffusion with volume fraction $f_{\mathrm{extra}}$ and diffusivity $D_{\mathrm{extra}}$. Under these assumptions, the SANDI model has an equivalent MD, given by

$$MD= f_{\mathrm{neurite}}\cdot\frac{1}{3} D_{\mathrm{neurite}} +\left( 1- f_{\mathrm{neurite}}-f_{\mathrm{extra}} \right)\cdot\mathrm{ADC}_{\mathrm{soma}}+ f_{\mathrm{extra}}\cdot D_{\mathrm{extra}}$$

Similarly, based on the above relation, the DKI-MD and SANDI-*f*_extra_ have a positive correlation due to

$$\frac{\partial MD}{\partial f_{\mathrm{extra}}}=-ADC_{\mathrm{soma}}+D_{\mathrm{extra}}>0,$$

with $D_{\mathrm{extra}}>ADC_{\mathrm{soma}}$ observed in most gray matter voxels.

Finally, we performed numerical simulations to demonstrate the relation between NODDI-ODI and DKI-AK. First, we synthesized direction-dependent diffusion signals based on the NODDI model and our diffusion MRI protocol (b-values = 800-3450 s/mm^2^) with varying intra-cellular volume fractions *f*_icvf_ from 0.2 to 1, isotropic volume fraction *f*_iso_ from 0 to 0.2, and orientation dispersion indices (ODI) from 0.05 to 1. Secondly, we fitted the DKI model to synthesized signals and estimated axial kurtosis (AK). Our simulation result demonstrated strong positive correlations between NODDI-ODI and DKI-AK.


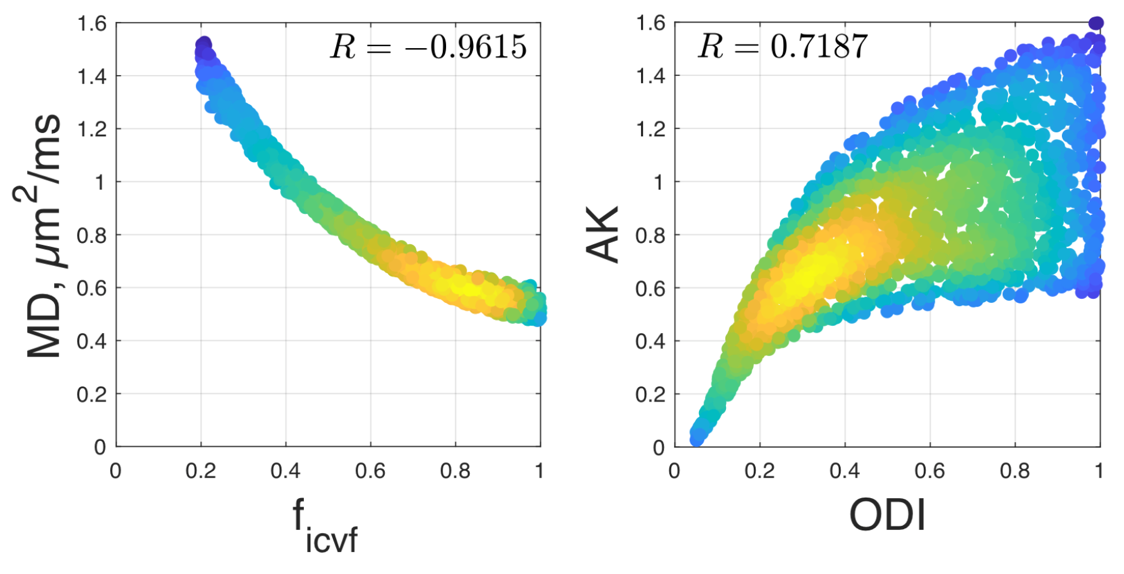


**Supplementary Figure S10:** Scatter plot shows a positive correlation between orientation dispersion index (ODI, from NODDI) and axial kurtosis (AK, from DKI). Spearman correlation coefficients (R) of 0.7187 quantifies their positive correlation.


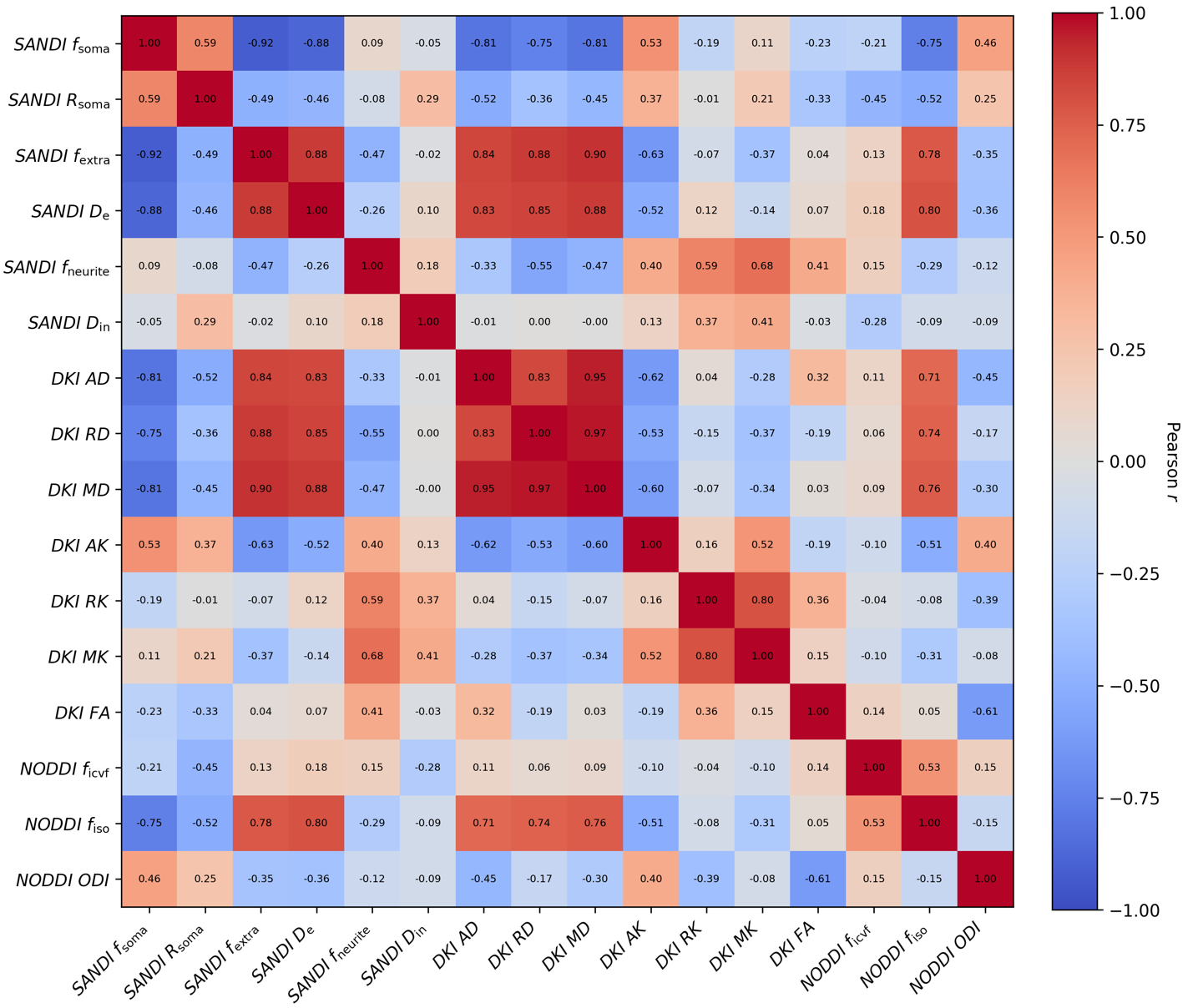


**Supplementary Figure S11.**Voxel‑wise Pearson correlation matrix between diffusion kurtosis imaging (DKI) metrics and biophysical model metrics derived from SANDI and NODDI in the bilateral hippocampus (averaged over **72 subjects**). Diffusion metrics including DKI (AD, RD, MD, AK, RK, MK, FA), SANDI (*f*_soma_, *R*_soma_, *f*_extra_, *D*_e_, *f*_neurite_, *D*_in_) and NODDI (*f*_icvf_, *f*_iso_, ODI) are incorporated in the correlation analysis. Consistent with theoretical predictions, positive correlations emerge between MD and both NODDI-*f*_iso_ and SANDI-*f*_extra_, and AK shows a strong positive association with ODI. These empirical relationships corroborate the analytical and simulation‑based analysis presented in **Supplementary Figure S10.**


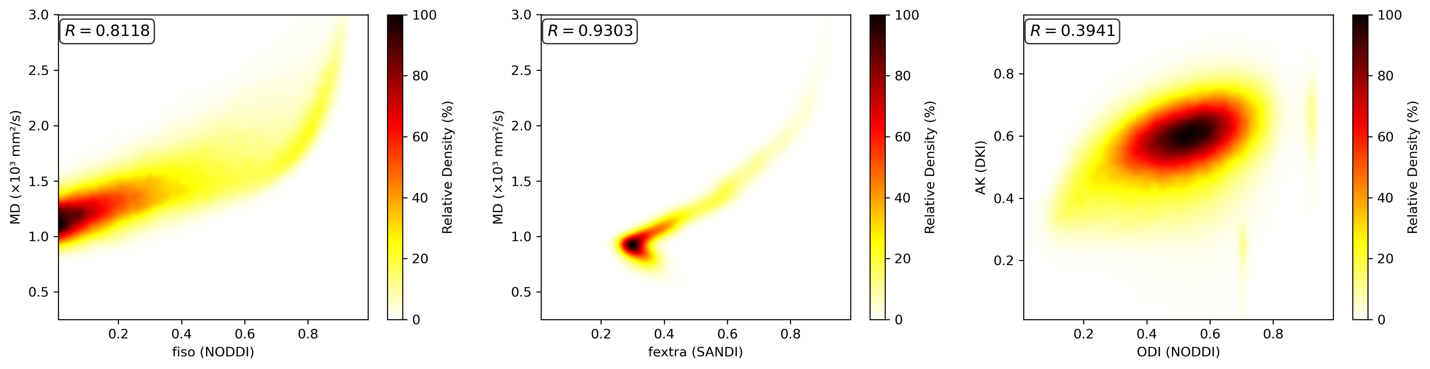


**Supplementary Figure S12.**Voxel-wise correlations between diffusion metrics: DKI-MD vs. NODDI-*f*_iso_, DKI-MD vs. SANDI-*f*_extra_ and DKI-AK vs. NODDI-ODI. Color indicates relative voxel density, with Pearson’s R shown in each panel. Analyses were performed across all voxels from both hippocampi in all subjects.
